# Efficacy and Effectiveness of SARS-CoV-2 Vaccines: A Systematic Review and Meta-Analysis

**DOI:** 10.3390/vaccines10030350

**Published:** 2022-02-23

**Authors:** Ramy Mohamed Ghazy, Rasha Ashmawy, Noha Alaa Hamdy, Yasir Ahmed Mohammed Elhadi, Omar Ahmed Reyad, Dina Elmalawany, Abdallah Almaghraby, Ramy Shaaban, Sarah Hamed N. Taha

**Affiliations:** 1 Tropical Health Department, High Institute of Public Health, Alexandria University, Alexandria 21561, Egypt; ramy_ghazy@alexu.edu.eg; 2 Department of Clinical Research, Maamoura Chest Hospital, Alexandria 21923, Egypt; mri.rasha.m.informatics19@alexu.edu.eg; 3 Department of Pharmacy Practice, Faculty of Pharmacy, Alexandria University, Alexandria 21521, Egypt; noha.alaaeldine@alexu.edu.eg; 4 Department of Public Health, Medical Research Office, Sudanese Medical Research Association, Khartoum P.O. Box 382, Sudan; hiph.yelhadi@alexu.edu.eg; 5 Department of Health Administration and Behavioral Sciences, High Institute of Public Health, Alexandria University, Alexandria 21561, Egypt; 6 Internal Medicine and Cardiology Clinical Pharmacy Department, Alexandria Main University Hospital, Alexandria 21526, Egypt; hiph.oreyad@alexu.edu.eg; 7 Primary Healthcare at Middle Medical District, Ministry of Health and Population, Alexandria 21523, Egypt; hiph.delmalawany@alexu.edu.eg; 8 Department of Cardiology and Angiology, Faculty of Medicine, Alexandria University, Alexandria 21524, Egypt; abdallah.aly@alexmed.edu.eg; 9 Department of Instructional Technology and Learning Sciences, Utah State University, Logan, UT 84321, USA; ramy.shaaban@usu.edu; 10 Forensic Medicine and Clinical Toxicology Department, Faculty of Medicine, Cairo University, Cairo 12613, Egypt

**Keywords:** SARS-CoV-2, efficacy, effectiveness, COVID-19 vaccine, systematic review, meta-anlysis, mortality

## Abstract

The coronavirus disease 2019 (COVID-19) pandemic has threatened global health and prompted the need for mass vaccination. We aimed to assess the efficacy and effectiveness of COVID-19 vaccines to prevent mortality and reduce the risk of developing severe disease after the 1st and 2nd doses. From conception to 28 June 2021, we searched PubMed, Cochrane, EBSCO, Scopus, ProQuest, Web of Science, WHO-ICTRP, and Google Scholar. We included both observational and randomized controlled trials. The pooled vaccine efficacy and effectiveness following vaccination, as well as their 95 percent confidence intervals (CI), were estimated using the random-effects model. In total, 22 of the 21,567 screened articles were eligible for quantitative analysis. Mortality 7 and 14 days after full vaccination decreased significantly among the vaccinated group compared to the unvaccinated group (OR = 0.10, ([95% CI, 0.04–0.27], I^2^ = 54%) and (OR = 0.46, [95% CI, 0.35–0.61], I^2^ = 0%), respectively. The probability of having severe disease one or two weeks after 2nd dose decreased significantly (OR = 0.29 [95% CI, 0.19–0.46], I^2^ = 25%) and (OR = 0.08 [95% CI, 0.03–0.25], I^2^ = 74%), respectively. The incidence of infection any time after the 1st and 2nd doses diminished significantly (OR = 0.14 [95% CI, 0.07–0.4], I^2^ = 100%) and (OR = 0.179 [95% CI, 0.15–0.19], I^2^ = 98%), respectively. Also, incidence of infection one week after 2nd dose decreased significantly, (OR = 0.04, [95% CI (0.01–0.2], I^2^ = 100%). After meta-regression, the type of vaccine and country were the main predictors of outcome [non-mRNA type, ß = 2.99, *p* = 0.0001; country UK, ß = −0.75, *p* = 0.038; country USA, ß = 0.8, *p* = 0.02]. This study showed that most vaccines have comparable effectiveness, and it is purported that mass vaccination may help to end this pandemic.

## 1. Introduction

Three novel coronaviruses have been discovered until the writing of this review. The first virus, named severe acute respiratory syndrome coronavirus 1 (SARS-CoV-1), was discovered in China in 2002 and caused severe acute respiratory syndrome. That same year, it led to more than 8000 infections and a 10% case fatality ratio (CFR) [1]. The second virus emerged in Saudi Arabia in 2012 and was called Middle East respiratory syndrome (MERS-CoV), with more than 2500 cases and a CFR of about 33% [2,3]. Following the appearance of SARS-CoV-1 and MERS-CoV, many vaccines were developed with live-attenuated, DNA-based, and recombinant viral vectors vaccines [4,5,6]. However, the development of clinical trials to test these postulated vaccines was abandoned when the outbreaks subsided due to the limited number of infections [7,8].

Late in 2019, a novel coronavirus called severe acute respiratory syndrome coronavirus 2 (SARS-CoV-2) emerged in Wuhan city, China [9]. The emerging virus causes a disease called coronavirus disease 2019 (COVID-19). Even though the great majority of SARS-CoV-2 infected patients have mild to moderate symptoms, the illness killed a considerable number of patients [10]. A hyperinflammatory process is known as “cytokine storm” is thought to be the cause of much of the serious disease associated with SARS-CoV-2 infection [11]. On 11 February 2022, 407.6 million got SARS-CoV-2 infection with 5.8 million deaths [12]. The CFR varied across countries from less than 0.1% to more than 25% [13].

The world is engaged in a fierce war against COVID-19; The United States Food and Drug Administration (FDA) [14] has granted Paxlovid from Pfizer an emergency use license for the treatment of mild-to-moderate COVID-19 in adults and pediatric patients (≥12 years of age) who are at high risk of progressing to severe COVID-19. Paxlovid is only accessible by prescription and should be started as soon as possible following a COVID-19 diagnosis and within five days of the onset of symptoms. 

The World Health Organization (WHO) has approved nine vaccines for emergency use up to December 2021. These include two RNA vaccines, Moderna (mRNA-1273) and Pfizer/BioNTech (BNT162b2); three non-replicating viral vectors, Janssen (Johnson & Johnson) (Ad26.COV2.S), Oxford/AstraZeneca (AZD1222), and Serum Institute of India Covishield (Oxford/AstraZeneca formulation); two protein subunits (NVX-CoV2373 and NovaVax); and inactivated virus techniques, Sinopharm (Beijing) BBIBP-CorV (Vero Cells) and Sinovac (CoronaVac) [15]. The vaccination process against COVID-19 started in December 2020 with the Pfizer-BioNTech, Moderna mRNA vaccines, and the Astra Zeneca/Oxford Chad Ox vaccines, as well as the Chinese Sinovac, inactivated SARS-CoV-2 and Russian Sputnik V adenovirus vaccines, and hundreds of vaccines at different stages of development and different mechanisms, including protein subunits with adjuvant, non-replicating viral vectors, RNA, virus-like-particles (VLP), DNA, inactivated, and live-attenuated virus [16,17]. On 23 August 2021, the FDA has approved the Pfizer-BioNTech vaccine to protect from COVID-19 for people above 16 years old. The vaccine’s previous emergency use authorization will continue for 12- to 15-year-olds [18]. Recently, Pfizer-BioNTech was approved for use among children aged 5–11 years [19]. In total, 61.7% of the population in the world has got at least one dose of COVID-19 vaccine. About 10.32 billion doses have been provided worldwide, and about 26.74 million are now administered each day [19]; however, only 10.6% of people living in low-income countries have received at least one dose. Until February 2022, less than 12% of Africans were fully vaccinated [20], while more than 62% of the population in Asia [21], 62% in South America, 63% in North America [22], and 70% in Europe were fully vaccinated [23].

The characteristics of an ideal vaccine are that it can be produced at a large scale with the lowest possible cost, that it is safe, easy to store and distribute, induces strong protection, has long-lasting neutralizing of antibody and T cell responses, and is equally suitable for any age and sex. Moreover, with the emergence of many variants of the COVID-19 virus, the vaccine also needs to be technically modifiable to deal with these emerging variants [24].

The major determinants of vaccine acceptance are vaccine safety and efficacy [25]. Most COVID-19 vaccines have mild side effects, such as pain at the site of injection, tiredness, headache, fever, or shivering for 1–2 days after vaccination. Very rare side effects include allergic reactions and blood clotting problems, the latter affecting a small number of people who had the Oxford/AstraZeneca vaccine [26]. Vaccine efficacy is defined as the degree to which a vaccine prevents disease, and possibly, also its transmission under ideal and controlled circumstances; this is determined by comparing a vaccinated group with a placebo group in a randomized controlled trial (RCT). Vaccine effectiveness also refers to how well the vaccine performs in the real world based on observational studies [27]. The aim of this systematic review and meta-analysis was to shed light on different studies evaluating the efficacy and effectiveness of COVID-19 vaccines after phase III trials.

## 2. Materials and Methods

This study was conducted in accordance with the Preferred Reporting Items of the Systematic Review and Meta-Analysis (PRISMA) checklist [28]. All steps were performed with strict compliance to the Cochrane Handbook of Systematic Review and Meta-Analysis [29]. Supplementary Table S1. Reference [1] is cited in Supplementary Materials.

**Inclusion and exclusion criteria**

All studies that met the following criteria were included:

- Reported COVID-19 vaccine efficacy (RCTs) or effectiveness (observational studies).
- Had a comparator group receiving either a placebo or another vaccine.
- The intervention group were either partially vaccinated (received only one dose of COVID-19 vaccine) or fully vaccinated.
- No restriction regarding country, race, gender, or age.
- We excluded abstract only letters to the editor, reviews, conference reports, study protocols, author responses, case reports, case series, and surveillance studies with no control group, in addition to any studies that had unreliable data for extraction or duplicates.

**Studied outcomes**

Primary outcomes:

- Efficacy and effectiveness of COVID-19 vaccines to prevent COVID-19 mortality.

Secondary outcomes:

- Efficacy and effectiveness of the vaccine to prevent severe disease.
- Efficacy and effectiveness of the vaccine to prevent SARS-CoV-2 infection within 7, 14, 21, and 28 days of the 1st dose.
- Efficacy and effectiveness of the vaccine to prevent SARS-CoV-2 infection any time after the 1st dose.
- Efficacy and effectiveness of the vaccine to prevent SARS-CoV-2 infection within 7 and 14 days of the 2nd dose, and 7,14 days after the 2nd dose.
- Efficacy and effectiveness of the vaccine to prevent SARS-CoV-2 infection any time after the 2nd dose.

**Operation cases definition**

Confirmed cases are persons who had a positive nucleic acid amplification test (NAAT), a person with a positive SARS-CoV-2 rapid diagnostic antigen test [30] and fulfill probable or suspected criteria of WHO case definitions, or a positive SARS-CoV-2 antigen (by rapid diagnostic test) asymptomatic patient but in close contact to probable or confirmed case [31].

Severe COVID-19: Adult patients categorized as having severe COVID-19, if matching one of the following criteria: oxygen saturation less than 90% in room air, a respiratory rate more than 30 breaths per minute, or had signs of severe respiratory distress [32].

Critical COVID-19: Adult patients with acute severe acute respiratory distress, septic shock, or any life-threatening condition needing critical care admission or mechanical ventilation [32].

Test negative cases control design: The best study design to detect risk factors of severe COVID-19 illness. In this study type, symptomatic COVID-19 patients are tested using a polymerase chain reaction (PCR) test, then categorized into cases (test positive patients) and controls (test-negative patients) [33].

**Search methods for identification of studies**

Electronic searches: The following databases were searched: Scopus, EBSCO, MEDLINE central/PubMed, Cochrane Central Register for Clinical Trials (CENTRAL), WHO International Clinical Trials Registry Platform (ICTRP), Web of Science (WOS), ProQuest Coronavirus database, and Google scholar. Search terms were determined and approved after the consultation with PubMed. The following keywords were used in our search, after adapting according to each database search strategy, (‘coronavirinae’ OR ‘coronaviridae infection’ OR ‘coronavirus disease 2019’ OR ‘coronavirus’ OR ‘coronavirus infection’) AND (Vaccin * efficacy * OR Vaccin * effective * OR Vaccin * immune *). We searched these databases to compile all studies on COVID-19 vaccination that were available up to 28 June 2021.

Searching other resources: In addition to searching the grey literature, a manual search of studies by checking reference lists of all eligible papers was undertaken to ensure that we did not miss any relevant study.

## 3. Data Collection and Extraction

Two independent reviewers searched the included databases (S.H. and R.M.G.). After which, all citations were exported to Endnote 20 to remove duplicates. Title and abstract screening was conducted by (R.A., A.A., N.H., D.M., and O.A.R.) and disagreement was resolved by (R.M.G). The inter-reviewer agreement was substantial (K = 0.8). Two reviewers (R.M.G. and S.H.) delineated accepted papers eligible for full-text screening. The two reviewers then extracted data related to patient characteristics and outcomes (authors, year of publication, country, inclusion, and exclusion criteria, when the study was conducted, study design, sample size, type of vaccine and number of doses, time point of analysis, primary and secondary outcomes). The Supplementary Materials of eligible articles were also reviewed for relevant data and the extracted data were checked and confirmed by a third author (Y.E.).

## 4. Data Analysis

Assessment of publication bias: We assessed publication bias through a visual inspection of the funnel plot and Egger’s test.

Quality assessment:

The risk of bias was assessed using two different tools according to the type of study: Cochrane risk of bias for randomized controlled trials (RoB2) [34] and the National Heart, Lung, and Blood Institute’s quality assessment tool for cohort, cross-sectional, and case-control studies [35]. D.M. and N.H. reviewed the quality of the studies and any disagreement was resolved by R.M.G. and S.H.

Effect size measurement:

We used the random-effects model to study the proposed outcomes due to the significant heterogeneity. The effect size was reported as odds ratio (OR) and 95% confidence interval level.

Assessment of heterogeneity:

- Visual inspection of the forest plot was carried out to analyze the consistency of intervention effects across the included studies. If the same intervention effect is estimated, there should be an overlap between the confidence intervals for each effect estimate on the forest plot. However, if the overlap is weak, or there are outliers, statistical heterogeneity is likely to be present.
- Statistical test for variation: heterogeneity was assessed by inspecting the forest plots to detect overlapping confidence intervals (CIs) and the I^2^ statistic used to denote levels of heterogeneity as defined in the Cochrane Handbook for Systematic Reviews of Interventions [30].

Heterogeneity was classified as follows:

- 0% to 40%: might not be important.
- 30% to 60%: may represent moderate heterogeneity.
- 50% to 90%: may represent substantial heterogeneity.
- 75% to 100%: considerable heterogeneity.

Sensitivity analysis: To conduct a sensitivity analysis, we recalculated the results of our meta-analysis K times, leaving out one study each time. This analysis also provides a classification for what is considered influential. We ordered studies in the plot via I^2^. Here we identified the studies with the highest heterogeneity as well as the final heterogeneity when these studies were removed.

Subgroup analysis: The included studies were divided into subgroups based on research design (RCT and observational), and the study outcomes were compared between the subgroups of studies.

Meta-regression: The outcome variable was the effect estimate (COVID-19 vaccine efficacy and effectiveness). The explanatory variables were study design (RCT/observational), type of vaccine (mRNA/not mRNA), and country where the study was conducted.

Statistical analysis: we used Review Manager (RevMan) version 5.4 and RStudio Desktop 2022.02.0+443 (meta package).

## 5. Results

### 5.1. Study Selection Process

**Figure 1:**
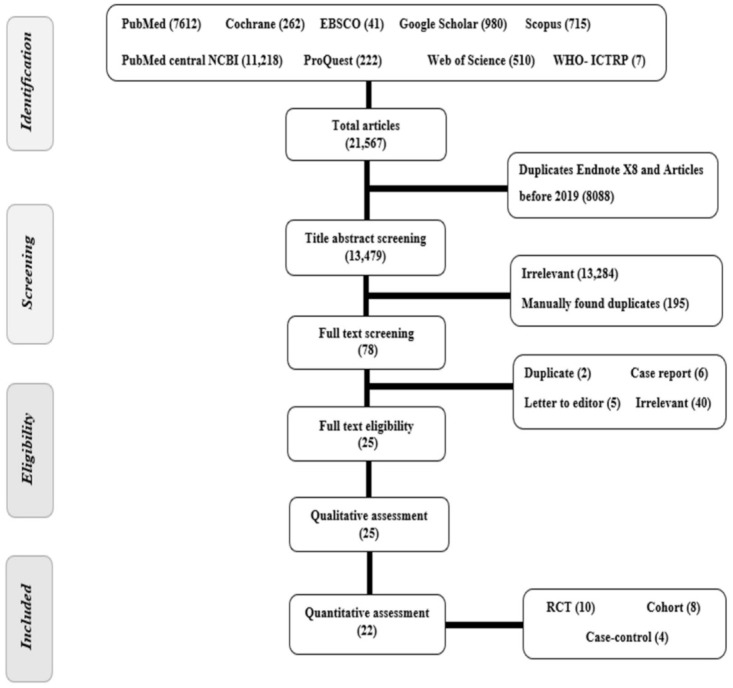
PRISMA flow chart of studies screened and a Summary of included studies in the qualitative analysis.

A total of 21,567 articles were found after searching nine different databases. Out of these, 8088 articles were excluded either because they were duplicates as found by Endnote X8 or because they were published before 2019. The title and abstract screening of 13,479 papers resulted in exclusion of 13,284 irrelevant papers and 195 manually found duplicates. A total of 78 articles were screened for eligibility. Finally, 22 papers were deemed eligible for the meta-analysis (Figure 1). List of papers rejected and causes of rejection are provided as Supplementary Table S2. References [2,3,4,5,6,7,8,9,10,11,12,13,14,15,16,17,18,19,20,21,22,23,24,25,26,27,28,29,30,31,32,33,34,35,36,37] are cited in Supplementary Materials.

In total, 25 studies were included in this review, 11 studies were RCTs [6,30,36,37,38,39,40,41,42,43,44] and 14 studies were observational studies [14,45,46,47,48,49,50,51,52,53,54,55,56,57] with total sample sizes ranged from 268 [48], to 6,538,911 subjects [49]. Among the included studies, two studies were conducted across countries [30,39]. We found that studies assessed the efficacy or effectiveness of vaccination after the 1st dose that ranged from 60 to 94.1% [14,30,37,40,43,45,46,47,48,50,52,53,54,56,57], while 18 studies assessed the efficacy or effectiveness of vaccination after 2nd dose that ranged from 21.1 to 100% [6,14,36,38,39,41,42,43,44,46,47,49,50,51,52,53,55,56]. Effectiveness or efficacy of BNT162b2 (Pfizer vaccine) was reported in 15 studies [14,39,42,45,46,47,48,49,50,51,52,54,55,56,57] while 6 studies addressed the AstraZeneca vaccine [38,41,43,52,54,57], 6 studies addressed the Moderna vaccine [14,37,51,53,55,56], and the Johnson and Johnson vaccine was studied in one study [30] (Table 1). 

### 5.2. Publication Bias

**Figure 2:**
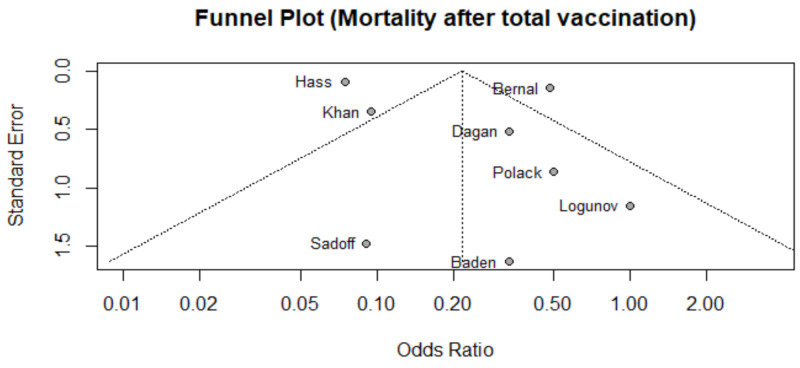
Funnel plot of studies that reported mortality after 2nd dose among vaccinated population.

We assessed publication bias of mortality associated with COVID-19 (primary outcome) through visual inspection of a funnel plot (Figure 2) and by conducting Egger’s test (t = 0.844, *p* = 0.43). We found that data was symmetric with low risk of publication bias.

## 6. Quality Assessment

**Figure 3:**
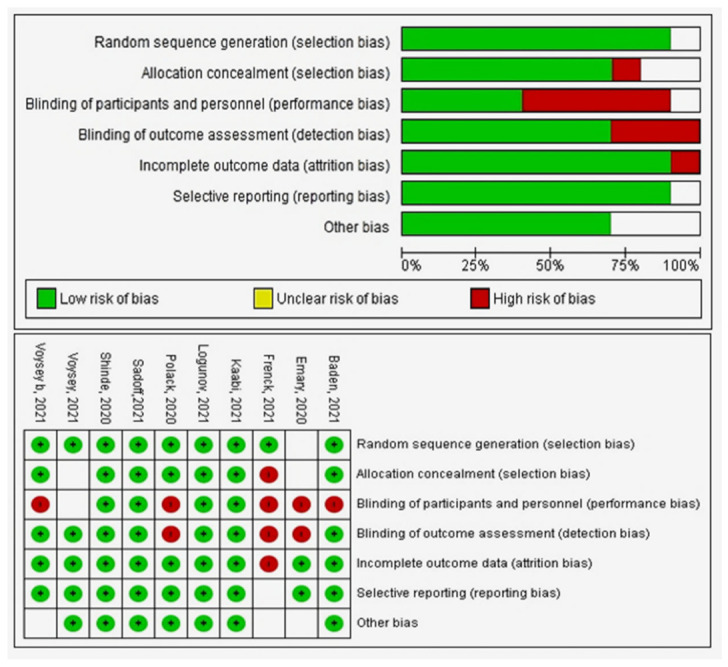
Quality assessment of RCT studies.

The quality assessment for the RCTs is presented in the summary of the risk of bias graph (Figure 3). The quality assessment of observational studies is included in Supplementary Table S3. References [38,39,40,41,42,43,44,45,46,47,48,49,50,51,52] are cited in Supplementary Materials.

### 6.1. Primary Outcome

#### Mortality after 7 and 14 Days after 2nd Dose

**Figure 4:**
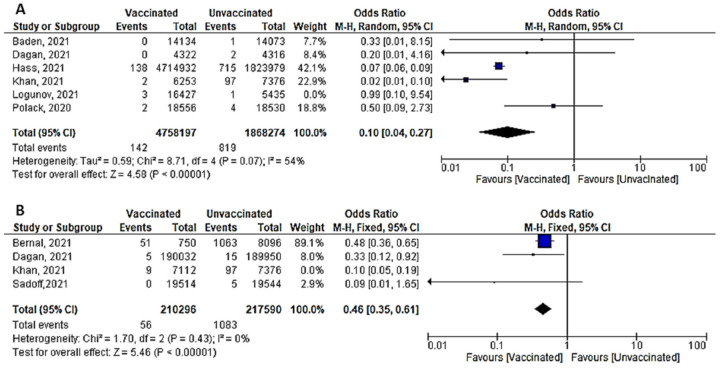
(**A**) Mortality 7 days after the 2nd dose of COVID-19 vaccination. (**B**) Mortality 14 days after 2nd dose of COVID-19 vaccination.

- A total of six studies (4 RCTs and 2 observational studies) assessed mortality due to COVID-19, 7 days after the 2nd dose of vaccination. Mortality among vaccinated subjects decreased significantly (OR = 0.14, [95% CI, 0.05–0.41], I^2^ = 63%). After conducting sensitivity analysis, the study of Logunov et al. [40] was removed, heterogeneity decreased to 54%; and the odds ratio became 0.10 [95% CI, 0.04–0.27], *p* < 0.001 (Figure 4A). It is important to note that in the study of Khan et al. [51] deaths due to COVID-19 were not separated from other causes of death.
- Four studies addressed mortality 14 days after 2nd dose, all of which were observational, except for the study by Sadoff et al. [30]. The overall mortality among vaccinated population decreased significantly (OR = 0.34, [95% CI, 0.26–0.44], I^2^ = 85%). After conducting a leave-one-out sensitivity analysis, the study of Khan et al. [51] was omitted. The observed heterogeneity dropped to 0% with an odds ratio of 0.46 [95% CI, 0.35–0.61], *p* < 0.001 (Figure 4B).
- Main findings: COVID-19 vaccination significantly decreased deaths 7 and 14 days after 2nd dose.

### 6.2. Secondary Outcomes

#### Severe COVID-19 Infection 7 Days after the 1st and 2nd Doses

**Figure 5:**
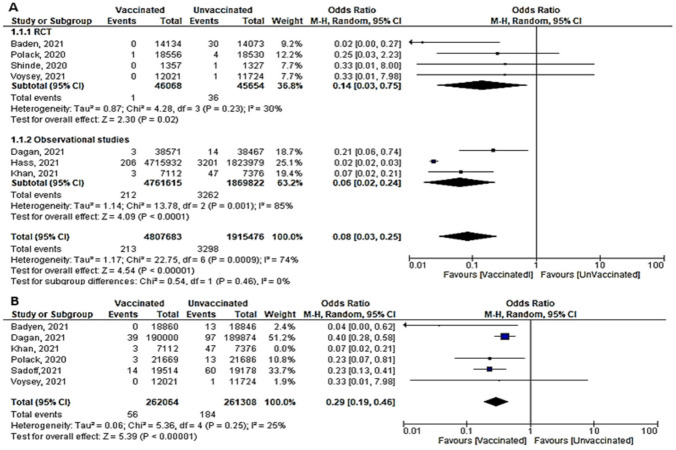
(**A**) Severe COVID-19 infection 7 days after 2nd dose of vaccination. (**B**) Severe COVID-19 infection 7 days after 1st dose of vaccination.

- Seven papers studied the incidence of severe COVID-19 infection 7 days after the 2nd dose. In total, 213 of 480,7683 vaccinated people developed severe COVID-19, compared to 3298 out of 191,5476 unvaccinated subjects. The odds ratio of developing severe COVID-19 among vaccinated subjects was 0.08 [95% CI, 0.03–0.25], I^2^ = 74%. After subgrouping the included studies into RCTs and observational, the heterogeneity dropped to 0%. The difference between observational and interventional studies was not significant (*p* = 0.46). The odds ratio of severe COVID-19 among vaccinated population in RCTs was 0.14 (95% CI, 0.03–0.75), I^2^ = 30%, *p* < 0.001 while in observational studies was 0.06 [0.02–0.24], I^2^ = 85%, *p* < 0.001. (Figure 5A).
- One week after the 1st dose, the odds ratio of developing severe COVID-19 after vaccination was OR = 0.21, [95% CI, 0.11–0.40], I^2^ = 61%. After omitting the study of Khan et al., [51] the odds ratio was 0.29 [95% CI, 0.19–0.46], I^2^ = 25% *p* < 0.001. (Figure 5B)
- Main findings: COVID-19 vaccination significantly decreased severe COVID-19 after vaccination with either 1 or 2 doses

### 6.3. Efficacy and Effectiveness of COVID-19 Vaccine in Reducing Infection Incidence after the 1st Dose

#### 6.3.1. Cases Reported within 7 Days of 1st Dose (Total Cases, Symptomatic and Asymptomatic)

- Two studies evaluated the efficacy and effectiveness of the COVID-19 vaccines in reducing SARS-CoV-2 infection (symptomatic and asymptomatic) within 7 days of the 1st dose. Dagan et al., [46] reported that 1965 of 596,618 vaccinated subjects got infection compared to 2362 of 596,618 unvaccinated subjects, OR = 0.83 [95% CI, 0.78–0.88]. 2362 of 596,618 unvaccinated subjects, OR = 0.83 [95% CI, 0.78–0.88]. Hall et al. [50] highlighted that the incidence of SARS-CoV-2 was 140 out of 20,641 unvaccinated subjects; it was lower than cases reported among unvaccinated (977 out of 2683), with OR = 0.01 [95% CI, 0.01–0.01]. Due to the significant heterogeneity, we could not pool the findings of these two outcomes.
- Bernal et al. [52] reported that 346 out of 864 vaccinated subjects versus 8988 out of 24,706 unvaccinated subjects got symptomatic SARS-CoV-2 infection, OR = 1.17 [95% CI, 1.02–1.34]. However, Dagan et al. [46] found that the COVID-19 vaccine had a protective effect, with an OR of 0.78 [95% CI, 0.72–0.84]. Among 596,618 vaccinated individuals, about 1103 subjects developed symptomatic SARS-CoV-2 infection, while among 596,618 unvaccinated individuals, about 1419 subjects developed symptomatic SARS-CoV-2 infection. Due to the significant heterogeneity, we could not pool the findings of these two outcomes.
- No studies reported asymptomatic cases within 7 days of the 1st dose.

#### 6.3.2. Cases Reported within 14 Days of 1st Dose:

**Figure 6:**
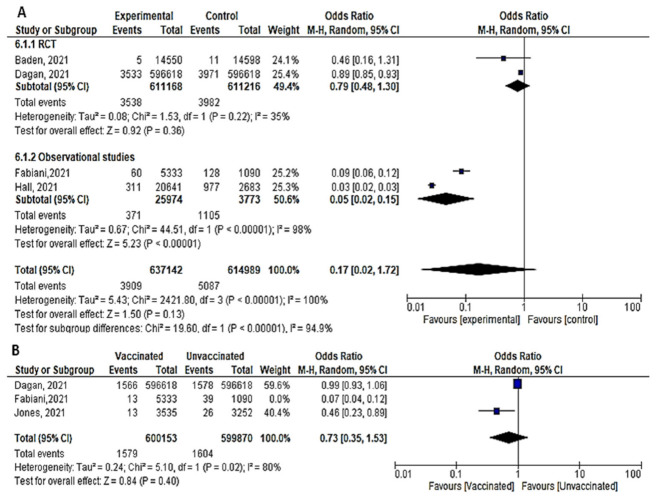
(**A**) Incidence of SARS-CoV-2 infection either symptomatic or asymptomatic within 14 days of 1st dose of vaccination. (**B**) Incidence of asymptomatic SARS-CoV-2 within 14 days of 1st dose of vaccination.

- Four studies assessed the efficacy and effectiveness of COVID-19 vaccines within 14 days of the 1st dose. In total, 3909 of 637,142 vaccinated subjects developed SARS-CoV-2 infection (symptomatic or asymptomatic) within 2 weeks after the 1st dose, compared to 5087 of 614,989 unvaccinated subjects. The odds ratio of getting SARS-CoV-2 infection decreased significantly (OR = 0.17 ([95% CI, 0.02–1.72], I^2^ = 100%). After we sub-grouped the included studies into RCTs and observational, the heterogeneity decreased to 94%. The difference between observational and interventional studies was significant (*p* = 0.001). The odds ratio of getting infection with SARS-CoV-2 in the RCTs was 0.79 [95% CI, 0.48–1.3], *p* = 0.36 while that of observational studies was 0.05 [95% CI, 0.02–0.15], *p* < 0.001. (Figure 6A).
- Bernal et al. [52] reported 958 symptomatic cases out of 1154 vaccinated subjects compared to 89 out of 8988 unvaccinated subjects, OR = 83.84 [95% CI, 68.07–103.26]. On the other hand, the protective effect of the COVID-19 vaccine was addressed by Dagan et al. [46] 1967 out of 596,618 vaccinated subjects versus 2393 out of 596,618 unvaccinated subjects OR = 0.82, [95% CI, 0.77–0.87]. On the same line, Fabiani et al. [47] reported that 47 out of 5333 vaccinated subjects versus 89 out of 1090 unvaccinated subjects developed symptomatic diseases, OR = 0.1, [95% CI, 0.07–0.14]. The results could not be pooled due to the significant heterogeneity.
- Three studies addressed the efficacy and effectiveness of vaccination in reducing the risk of having asymptomatic COVID-19 within 14 days of the 1st dose (OR = 0.23 [95% CI, 0.06–1.63], I^2^ = 97%). After conducting a leave-one-out sensitivity analysis, the study by Fabiani et al., [47] was omitted. The probability of getting asymptomatic infection did not reduce significantly. (OR = 0.73 [95% CI, 0.35–1.53], I^2^= 80%), *p* < 0.02. (Figure 6B).
- Main finding: incidence of SARS-CoV-2 infection in observational studies decreased significantly after vaccination within 14 days of 1st dose.

#### 6.3.3. Cases Reported within 21 Days of 1st Dose

**Figure 7:**
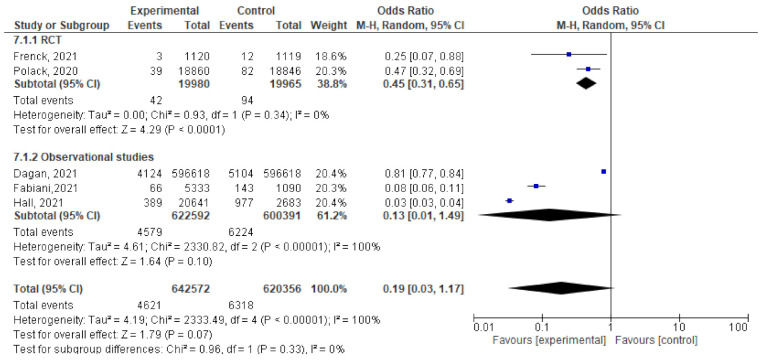
Incidence of SARS-CoV-2 infection either symptomatic or asymptomatic within 21 days after the 1st dose of vaccination.

- Five studies assessed the efficacy and effectiveness of COVID-19 vaccines within 21 days of the 1st dose. In total, 4621 of 642,572 vaccinated subjects developed SARS-CoV-2 infection (symptomatic and asymptomatic) within 3 weeks of the 1st dose, compared to 6318 out of 620,356 unvaccinated subjects. The odds ratio of having infection was 0.19 [95% CI, 0.03–1.17], I^2^ = 100%. After subgrouping the included studies into RCTs and observational, the heterogeneity dropped to 0%. The difference between observational and interventional studies was insignificant (*p* = 0.33). The odds ratio of infection with COVID-19 in the RCTs was 0.45 [95% CI, 0.31–0.65], *p* < 0.001, while the OR in observational studies was 0.13 [95% CI, 0.01–1.49], *p* < 0.001 (Figure 7).
- For Dagan et al. [46], the odds ratio of catching symptomatic SARS-CoV-2 after vaccination was 0.73 [95% CI, 0.69–0.77]. Of the 596,618 vaccinated subjects, 2250 developed symptomatic SARS-CoV-2 infection, compared to 3079 out of 596,618 unvaccinated subjects. Fabiani et al. [47] reported that 51 out of 53,333 vaccinated subjects developed symptomatic disease compared to 97 out of 1090 unvaccinated subjects, OR = 0.1 (95% CI, 0.07–0.14). The results could not be pooled due to the significant heterogeneity.
- Only the study by Fabiani et al. [47] addressed the effectiveness of COVID-19 against asymptomatic infection three weeks after the 1st dose. In total, 15 out of 5333 vaccinated subjects developed asymptomatic SARS-CoV-2 compared to 46 out of 1090 unvaccinated subjects, OR = 0.06 [95% CI, 0.04–0.12].
- Main findings: incidence of SARS-CoV-2 infection within 21 days of the 1st dose of vaccination reduced significantly.

#### 6.3.4. Cases Reported within 28 Days of 1st Dose

- The total number of cases (symptomatic and asymptomatic) reported 28 days after the 1st dose was reported in two studies; Dagan et al. [46] documented 4405 confirmed cases of 596,618 vaccinated individuals compared to 5775 confirmed cases of 596,618 unvaccinated subjects. Hall et al. [50] diagnosed 427 cases of 20,641 vaccinated subjects, and 977 cases of 2683 unvaccinated subject. The odds ratios were 0.76 [95% CI, 0.73–0.79] and 0.04 [95% CI, 0.03–0.4], respectively. The research team could not pool the odds ratios due to the significant heterogeneity.
- No studies reported asymptomatic or symptomatic cases within 28 days of the 1st dose.

#### 6.3.5. Cases Reported Any Time after 1st Dose

**Figure 8:**
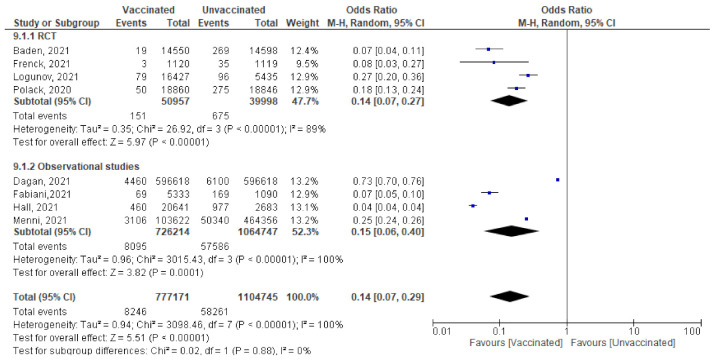
Incidence of all SARS-CoV-2 cases after 1st dose of vaccination.

- Among 777,171 vaccinated subjects with a single dose of COVID-19 vaccine, 8246 got SARS-CoV-2 infection (symptomatic or asymptomatic) compared to 58,261 of 1,104,745 unvaccinated subjects (OR = 0.14 [95% CI, 0.07–0.4], I^2^ = 100%). After subgrouping based on the study design, the heterogeneity dropped to 0%, the odds ratio of RCTs was 0.14 [95% CI, 0.07–0.27], *p* < 0.001 and of observational studies was 0.15 [95% CI, 0.06–0.4] *p* < 0.001. The difference between subgroups was not significant, *p* = 0.88. (Figure 8)
- Main findings: incidence of SARS-CoV-2 infection any time after the 1st dose decreased significantly among vaccinated compared to unvaccinated.

### 6.4. Efficacy/Effectiveness of the 2nd Dose

#### 6.4.1. Cases Reported within 7 days of 2nd Dose

**Figure 9:**
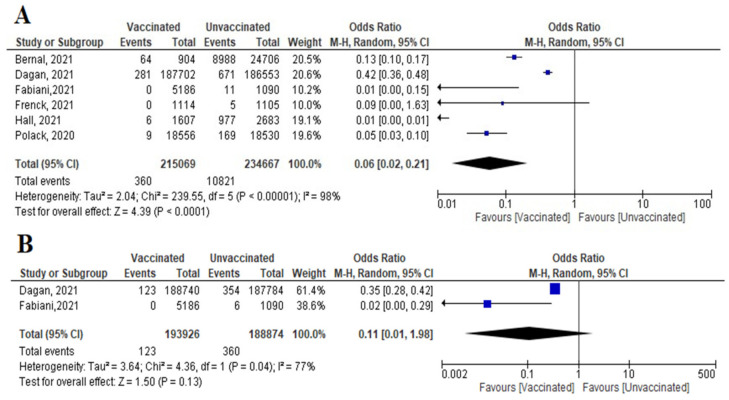
(**A**) Incidence of SARS-CoV-2 infection either symptomatic or asymptomatic within 7 days of 2nd dose of vaccination. (**B**) Incidence symptomatic SARS-CoV-2 within 7 days of 2nd dose of vaccination.

- Six studies reported COVID-19 cases within 7 days of vaccination; two were RCTs and four were observational studies. The number of cases reported decreased significantly among the vaccinated versus non-vaccinated population (OR = 0.06 (95% CI, 0.02–0.21), I^2^ = 98%). We conducted a meta-regression to explain this substantial heterogeneity, using the type of vaccine and country as predictors [mRNA and non-mRNA]. The output of the developed meta-regression model explained 100.0% of the heterogeneity (variability in our data); [mRNA type, β = −3.33, *p* = 0.028; country UK, β = −3.7, *p* = 0.025; country USA, β = −2.03, *p* = 0.037: Israel was the reference country] (Figure 9A). Meaning that vaccination using mRNA decreases the incidence of SARS-CoV-2 infection by 3.33 compared to other vaccines, and vaccination in developed countries like UK and USA decreased the incidence of infection by 3.7 and 2.03 times less than the reference country, respectively.
- Incidence of symptomatic SARS-CoV-2 infection did not decrease significantly among vaccinated compared to unvaccinated (OR = 0.11 [95% CI, 0.01–1.98], I^2^ = 77%), *p* = 0.13 (Figure 9B).
- No studies reported asymptomatic cases within 7 days of the 2nd dose.
- Main findings: COVID-19 vaccination decreased incidence of SARS-CoV-2 infection within 7 days of 2nd dose. The main predictor of vaccine effectiveness/efficacy were the country where the vaccine was supplied and the type of vaccine.

#### 6.4.2. Cases within 14 Days of 2nd Dose

**Figure 10:**
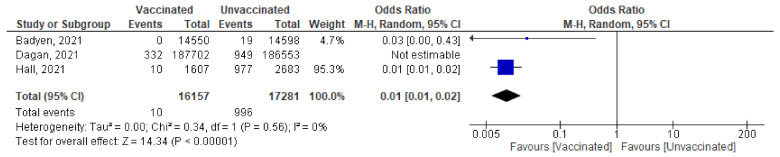
Incidence of SARS-CoV-2 infection either symptomatic or asymptomatic within 14 days of 2nd dose of vaccination.

- Three studies highlighted the number of new cases (symptomatic and asymptomatic) reported within 14 days of 2nd dose. Baden et al. [37] reported no cases among 14,550 vaccinated subjects compared to 19 cases out of 14,598 unvaccinated subjects. Dagan et al. [46] reported 332 confirmed cases out of 187,702 vaccinated subjects and 949 cases out of 186,553 unvaccinated subjects. Hall et al. [50] reported 10 cases out of 1607 vaccinated subjects and 977 cases out of 2683 unvaccinated subjects. Pooling of these findings showed significant reduction in incidence of reported cases (OR = 0.05 [95% CI, 0.0–1.34], I^2^ = 99%). After conducting leave-one-out sensitivity analysis, the study by Dagan et al. [46] was omitted, the vaccine was effective in reducing the number of cases reported within 14 days of the 2nd dose (OR = 0.01 [95% CI, 0.01–0.02], I^2^ = 0%), *p* < 0.001 (Figure 10).
- No studies reported asymptomatic or symptomatic cases within 14 days of 2nd dose.
- Main findings: incidence of SARS-CoV-2 infection within 14 days of the 2nd dose decreased significantly after vaccination.

#### 6.4.3. Cases Reported 7 Days after 2nd Dose

**Figure 11:**
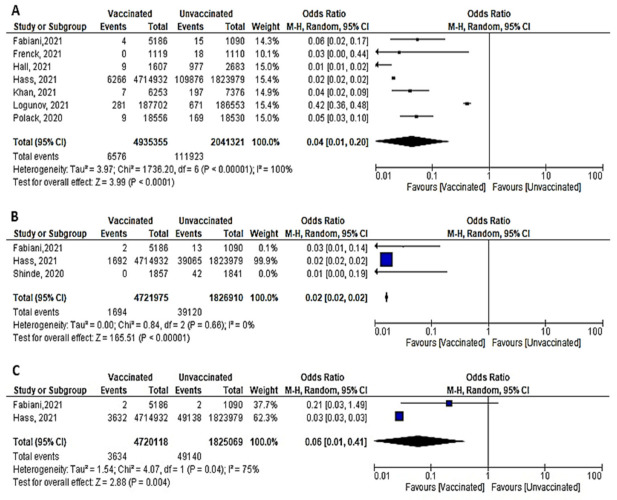
(**A**) Incidence of SARS-CoV-2 infection either symptomatic or asymptomatic after 7 days of 2nd dose of vaccination. (**B**) Incidence of symptomatic SARS-CoV-2 after 7 days of 2nd dose of vaccination. (**C**) Incidence of asymptomatic SARS-CoV-2 after 7 days of 2nd dose of vaccination.

- Among 4,935,355 vaccinated subjects, the number of confirmed SARS-CoV-2 infection (symptomatic and asymptomatic) 7 days after 2nd dose was 6576, while 111,923 confirmed cases were reported among 2,041,321 unvaccinated subjects. The OR was 0.04 [95% CI, 0.01–0.2], I^2^=100, *p* < 0.001, indicating that COVID-19 vaccines had a protective effect. We were able to explain 100% of this heterogeneity by conducting meta-regression analysis. Vaccine type and country were significant predictors of response; [non-mRNA vaccine (β = 2.99, *p* = 0.0001): mRNA is the reference type, country UK (β = –0.75, *p* = 0.038), country USA (β = 0.8, *p* = 0.02); reference country was Israel] (Figure 11A). In this case, non-mRNA (the highest OR) vaccine and vaccination in the USA increased the risk of SARS-CoV-2 infection by 2.99 and 0.8 times, respectively. On the other hand, vaccination in the UK decreased risk of SARS-CoV-2 infection by 0.75 comparing to reference country (Israel).
- COVID-19 vaccines were effective in preventing symptomatic COVID-19 infection 7 days after the 2nd dose (OR = 0.02 [95% CI, 0.02–0.02], I^2^ = 0%), *p* < 0.001 (Figure 11B).
- Two studies (recruiting 4,720,118 vaccinated subjects and 1,825,069 unvaccinated subjects) assessed asymptomatic SARS-CoV-2 infection after 7 days of vaccination. Fabini et al. [47] diagnosed two cases among vaccinated and non-vaccinated subjects with a statistically non-significant odds ratio (OR = 0.21, [95% CI, 0.03–1.49]). Meanwhile, Hass et al. [49] reported 3632 asymptomatic cases among vaccinated subjects and 49,138 among unvaccinated subjects (OR = 0.03, [95% CI, 0.03–0.03]) (Figure 11C). Vaccination was protective against asymptomatic infection 7 days after the 2nd dose (OR = 0.06 [95% CI, 0.01–0.41), I^2^ = 75%), *p* = 0.04.
- Main findings: incidence of SARS-CoV-2 (symptomatic and asymptomatic) reported 7 days after the 2nd dose decreased significantly.

#### 6.4.4. Cases Reported 14 Days after 2nd Dose

**Figure 12:**
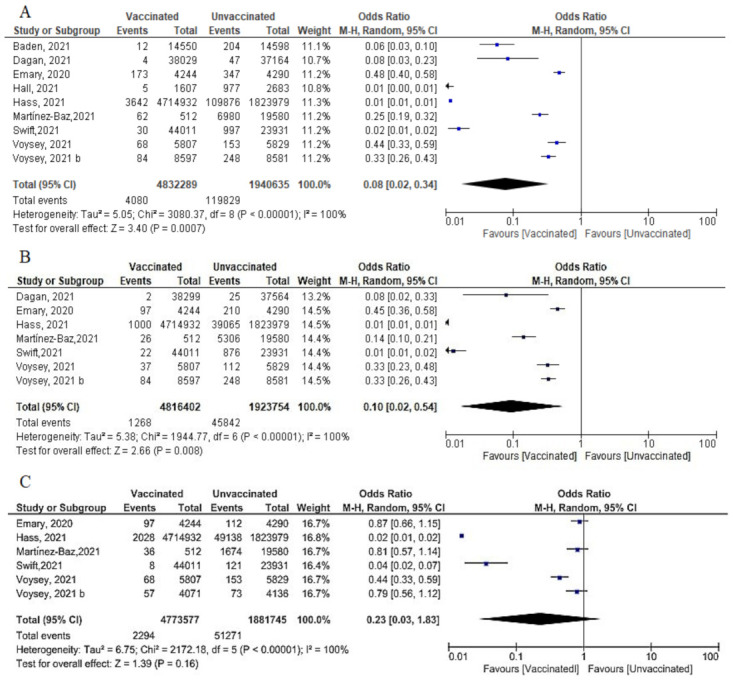
(**A**) Incidence of SARS-CoV-2 infection either symptomatic or asymptomatic after 14 days of 2nd dose of vaccination. (**B**) Incidence of symptomatic SARS-CoV-2 after 14 days of 2nd dose of vaccination. (**C**) Incidence of asymptomatic SARS-CoV-2 after 14 days of 2nd dose of vaccination.

- We pooled nine studies that addressed the total number of symptomatic or asymptomatic cases confirmed 14 days after the 2nd dose of vaccination. The total number of cases was 4080 out of 4,832,289 vaccinated subjects and 119,829 out of 1,940,635 unvaccinated subjects. The vaccine had a protective effect against getting SARS-CoV-2 infection 14 days after the 2nd dose (OR = 0.08, [95% CI, 0.02, 0.34], I^2^ = 100%), *p* < 0.001. Thus, we carried out a meta-regression analysis to understand the main predictors of this heterogeneity. Again, the type of vaccine and the country were responsible for 88.21% of the heterogeneity; [non-mRNA vaccine β = 3.519, *p* = 0.004; country Spain β = 2.6256, *p* = 0.028: Israel was the reference country) (Figure 1A).
- Seven studies reported the incidence of symptomatic infection 14 days after the 2nd dose. Vaccination had a protective effect against symptomatic infection. The odds among vaccinated subjects was (0.10 [95% CI, 0.02–0.54], I^2^ = 100%), *p* < 0.001. To further explain this substantial heterogeneity, we performed meta-regression analysis that explained 100% of the heterogeneity using type of vaccine and country as predictors; [non-mRNA type, β = 3.16, *p* = 0.0044; country Spain β = 2.7, *p* = 0.0048; Israel is the reference country] (Figure 12B).
- Regarding asymptomatic cases after 14 days of the 2nd dose, 2294 of 4,773,577 vaccinated subjects compared to 51,271 of 1,881,745 unvaccinated subjects developed asymptomatic infection. (OR = 0.23 [95% CI, 0.03–1.83], I^2^ = 100%.), *p* < 0.001. Meta-regression was performed and explain 96.29% of heterogeneity, using type of vaccine and the country as predictors, [non-mRNA type, β = 2.9, *p* = 0.033; country Spain β = 3.96, *p* = 0.016: reference Israel]. (Figure 12C).
- Main findings: incidence of SARS-CoV-2 cases (total cases and asymptomatic cases) two weeks after the 2nd dose was significantly reduced.

#### 6.4.5. Cases Reported 7–14 Days after 2nd Dose

Dagan et al. [46] reported 51 cases out of 108,529 vaccinated subjects and 278 cases out of 107,209 unvaccinated subjects got SARS-CoV-2 infection 7–14 days after the 2nd dose of vaccination OR = 0.18 [95%CI, 0.13–0.24]. Meanwhile, Hall et al. [50] reported 4 cases out of 1607 vaccinated subjects and 977 out of 2683 unvaccinated subjects, OR = 0.0 (95% CI, 0.0–0.01). The result could not be pooled due to substantial heterogeneity I^2^ = 99%.

#### 6.4.6. All Cases Reported after the 2nd Dose

**Figure 13:**
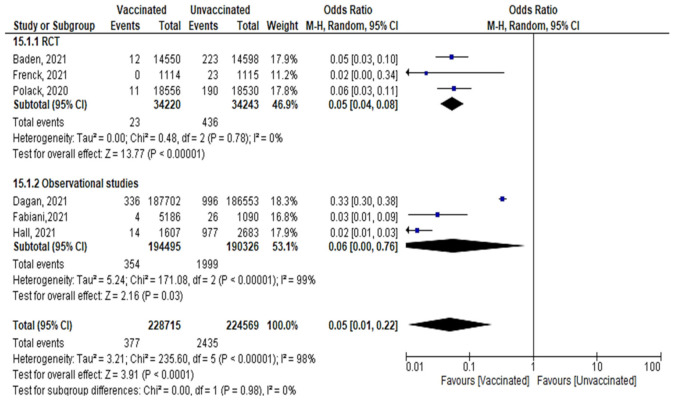
All cases of confirmed SARS-CoV-2 infection reported after 2nd dose of vaccination regardless of the duration.

- A total of 377 confirmed cases out of 228,715 vaccinated subjects, and 2435 cases out of 224,569 unvaccinated subjects were reported after the 2nd dose, (OR = 0.179 [95% CI, 0.15–0.19], I^2^ = 98%). The test for subgroup differences suggested that there was no statistically significant subgroup effect (*p* = 0.98), meaning that the type of study did not significantly modify the efficacy and effectiveness of vaccination. Vaccination decreased the number of cases regardless of the study design, although the protective effect was greater in RCT than in observational studies. There was no heterogeneity between the results of the RCT studies, while the heterogeneity of observational studies was I^2^ = 99% (Figure 13).
- Main findings: vaccination against SARS-CoV-2 decreased incidence of infection after the 2nd dose regardless of the duration.

## 7. Discussion

The aim of vaccine development is to provide a weapon that protects people from getting infected or becoming a source of transmission. By the end of 2020, several COVID-19 vaccines had become available for use across the world, with over 40 different vaccines in human trials, and over 150 in preclinical trials. An updated list of vaccine candidates under evaluation is maintained by the WHO [58]. Although some of the vaccines were approved for emergency use by the FDA in the USA and the respective health departments of other countries across the world, the efficacy and effectiveness should be periodically assessed due to the ongoing antigenic drift. It is worth noting that while vaccinations are still being administered worldwide, the vaccinated population (received at least one dose of vaccine) represents around three-fifth of the entire population [59], with safety and effectiveness representing the main concern and points of hesitation for many people [25]. Another main concern affecting vaccination coverage is COVID-19 vaccine inequity; vaccine supply will have a long-term and severe impact on socioeconomic recovery in low-and lower-middle-income countries (LMIC) unless immediate action is taken to increase supply and provide equal access for all countries. If LMIC had similar vaccination rates as high-income countries (HIC), an acceleration in scaling up manufacturing and providing adequate vaccine doses might have added costs. A high price per COVID-19 vaccine dose in comparison to other vaccines, as well as delivery costs, including those for the health workforce surge, could put a huge strain on fragile health systems, undermining vaccination programs and essential health services, and causing alarming spikes in measles, pneumonia, and diarrhea [60].

Several studies recruited different numbers of participants to study various outcomes with variable endpoints providing different doses of vaccine with variable intervals. Studying the efficacy and effectiveness of several types of COVID-19 vaccines in reducing mortality and severity were the focus of this meta-analysis aiming to provide strong evidence to decision-makers in health policy sectors to deal with the ongoing pandemic.

We reviewed a total of 22 articles to study the desired outcomes, among which 10 were RCTs, 4 were case-control, and 8 were cohort studies. The highest number of recruited subjects in a single study was 6,538,911 [49], while the smallest number was 268 subjects [48].

### 7.1. Mortality and Severe COVID-19

Based on the findings of this meta-analysis, the mortality related to COVID-19 two weeks after vaccination was significantly decreased (OR = 0.46, [95% CI, 0.35–0.61], I^2^ = 0%). Similarly, mortality one week after vaccination dropped significantly (OR = 0.10, [95% CI, 0.04–0.27], I^2^ = 54%). In RCTs, the odds ratio for severe COVID-19 was 0.14 [95% CI, 0.03–0.75], I^2^ = 30%), whereas in observational studies, the odds ratio was 0.06 [0.02–0.24], I^2^ = 85%. In the same line, the odds ratio of having severe COVID-19 after the 1st dose was 0.15 [0.10–0.25], I^2^ = 26%. Analyzing our results, different studies reported a significant reduction in SARS-CoV-2 infection, hospitalizations, and fatalities among those who had been fully vaccinated compared to those who had not been fully vaccinated [61,62,63]. Fiolet et al. [64] recently published a review on different COVID-19 vaccines effectiveness; when the strain was not sequenced, the effectiveness of the mRNA vaccination against hospitalization and mortality was over 87–94%. Similarly, inactivated viral COVID-19 vaccine (CoronaVac) was extremely effective against hospitalization (87.5%) and death (86.3%). In addition, if a breakthrough occurs in a vaccinated individual, the events are usually less severe than in an unprotected person [65]. Similarly, a recently published meta-analysis highlighted that the BNT162b2 and mRNA-1273 vaccines had the best effectiveness in preventing symptomatic COVID-19. The efficacy of comparing different vaccines in preventing serious illness was not different. Moreover, there was no difference in the efficacy of vaccinations to prevent symptomatic COVID-19 among the elderly [66]. Unfortunately, this protective effect wanes with time—5 months or more after vaccination—and vaccine effectiveness decreased against hospitalization and deaths (80.0 and 84.8% with the ChAdOx1-S) and (91.7% and 91.9% with BNT162b2), respectively [67].

### 7.2. Infection after Vaccination

It is worthy to note that Alagoz et al. [68] hypothesized that if there is a strong adherence to non-pharmacological interventions in the community, the controllable spread of SARS-CoV-2 can be reached sooner than when a substantial part of the population gets vaccinated (e.g., 70–80%). In the current study, COVID-19 vaccines effectively reduced the incidence of symptomatic and asymptomatic infection. On the same lines, the WHO reported that unvaccinated persons account for the great majority of the current SARS-CoV-2 infection [65]. Virus-neutralizing antibodies are principally responsible for the protection provided by presently available vaccinations. These antibodies often inhibit the virus’s binding with its cellular receptor or prevent the virus from undergoing the conformational changes essential for fusion with the cell membrane [69] We found that vaccination against COVID-19 decreased the number of cases reported within a week of the 2nd dose (OR = 0.06 (95% CI, 0.02–0.21), I^2^ = 98%). Type of vaccine and country where study was conducted were the main predictors of vaccine efficacy and effectiveness. Similarly, the total number of cases diagnosed within 14 days of the 2nd dose decreased significantly, (OR = 0.01 [95% CI, 0.01–0.02], I^2^ = 0%). In terms of cases reported 7 days after 2nd dose, the total number of cases decreased significantly with vaccination (OR = 0.03 [95% CI, 0.02–0.05], I^2^ = 73%). About 100% of this heterogeneity was explained by meta-regression (vaccine type and country). Regarding symptomatic cases diagnosed 7 days after the 2nd dose, COVID-19 vaccine was effective in reducing the number of symptomatic cases in comparison to placebo or control group (OR = 0.02 [95% CI, 0.02–0.02], I^2^ = 0%). The odds ratio of cases reported 14 days after the 2nd dose among vaccinated versus unvaccinated subjects was OR = 0.08, [95% CI, 0.02-0.34], I^2^ = 100%). Confirmed cases reported after the 1st and 2nd dose regardless of the duration decreased significantly, OR = 0.14 (95% CI, 0.07–0.4) I^2^ = 100% and 0.18 (95% CI, 0.15–0.19), I^2^ = 98%, respectively.

In the same vein, many reviews addressed vaccine effectiveness and efficacy. Pormohammad et al. [70] included 25 studies in phase II/III RCTs, the efficacy of mRNA-based and adenovirus-vectored COVID-19 vaccines was 94.6% and 80.2%, respectively. After 3 weeks of vaccinations, the adenovirus-vectored vaccine had the maximum efficacy against receptor-binding domain (RBD) antigen after the 1st and 2nd doses (97.6% and 98.2% respectively). Similarly, a review of phase III studies showed a significant increase in neutralizing antibodies with the 2nd dose of the vaccine [71]. However, it was also advised that when vaccine supply is scarce, countries should vaccinate with a single dose. This may provide better overall protection in the population than vaccinating half the number of individuals with both doses [72].

Many factors can explain the observed difference in efficacy and effectiveness of the COVID-19 vaccines. The Center for Disease Control and Prevention [73] demonstrated that in the real-world, vaccine effectiveness can be affected by several factors, including population host factors (e.g., those who were not included in clinical trials) and virus factors (e.g., variants) as well as programmatic factors (e.g., adherence to dosing schedules or vaccine storage/handling) [74]. Thompson et al. [75] reported that under real-world conditions, complete immunization (14 days after 2nd dose) was 90% effective against SARS-CoV-2 infection, while partial immunization (14 days after 1st dose but before 2nd dose) was 80% effective. In addition, the effectiveness of vaccination varied according to the types of vaccine; Pilishvili et al. [76] stated that vaccine effectiveness for Pfizer–BioNTech and Moderna were 77.6% (95.6% CI, 70.9–82.7) and 88.9% (95.9% CI, 78.7–94.2) after the 1st dose and were 88.8% (95% CI, 84.6–91.8) and 96.3% (95.3–98.4) after the 2nd dose, respectively. Of note, when the SARS-CoV-2 Delta variant became prevalent, the percentage of completely vaccinated people who got SARS-CoV-2 infection grew higher than predicted [63]. The effectiveness of the mRNA vaccine against COVID-19 was 88–100% against Alpha, 76–100% against Beta/Gamma, 47.3–88% against Delta, and 89–100% when the SARS-CoV-2 strain was not sequenced. Oxford/AstraZeneca (AZD1222) was 74.5% effective against Alpha and 67% effective against Delta. CoronaVac was effective against the Alpha/Gamma/D614G strain in 36.8–73.8% of cases [64].

Unfortunately, new data consistently demonstrated that vaccine efficacy against SARS-CoV-2 infection declines with time following immunization [77]. It is worth noting that according to a recently published systematic review and meta-analysis, immunization efficacy against severe COVID-19 infection dropped by around 8% (95% CI, 4–15) during the 6-months period in all age groups. Over the same time, vaccine efficacy against serious illness declined by around 10% (95% CI, 6–15%) in individuals over the age of 50. Vaccine efficacy against symptomatic illness fell by 32% (95% CI, 11–69%) in individuals over the age of 50 [78]. Consequently, WHO has already recommended administering a booster dose of vaccine to people aged 60 years or older as part of the main series to strengthen initial protection [65]. Therefore, people should adhere to public health and social measures even though they have received vaccines to avoid COVID-19 infection and its consequences [79].

### 7.3. Strengths and Limitations

This systematic review has some limitations. First, there is no evidence of the long-term effectiveness of the vaccine. Due to the urgency of vaccine development, most trials only followed up participants for 28 days after vaccination. Second, this metanalysis cannot give solid evidence on the efficacy and effectiveness of COVID-19 vaccines on the variant strain B.1.351. This variant strain can escape neutralizing relevant antibodies. Consequently, more studies need to be conducted to assess the effectiveness and efficacy of COVID-19 vaccines against variants of concern like delta strain and omicron [64]. Third, due to scarce of literature, we neither included all approved vaccines nor all age groups (elderly, adolescents, and children). 

The points of strength in this systematic review are that we did not include preprinted documents, studies that were not peer-reviewed, and studies with missing data. Due to the scarcity of RCTs, observational studies were included, as were retrospective case analyses. Animal studies were excluded, and we did not have lingual restrictions. Our analysis has identified numerous critical components to consider when planning a real-world efficacy trial of COVID-19 vaccinations, such as the appropriate study design, study population, outcome, and period for follow-up. The majority of studies identified were from HICs, frequently utilizing national databases (which may not exist or may be of lower quality in LMICs), and the vast majority assessed mRNA vaccines, which are more prevalent in HICs. These findings highlight the need for pressing for real-world efficacy studies on all licensed COVID-19 vaccines in a variety of LMIC contexts using different study designs.

## 8. Conclusions

This systematic review and meta-analysis summarized the results of clinical trials related to the COVID-19 vaccines, showing that most vaccines had comparable effectiveness and efficacy. It is believed that vaccination can effectively reduce COVID-19 related deaths and severe cases. The incidence of COVID-19, either symptomatic or asymptomatic, decreased significantly after vaccination by one or two doses. However, in the light of the ongoing appearance of novel variants, the efficacy/effectiveness of vaccination against COVID-19 infection needs to be re-assessed.

## Figures and Tables

**Table 1:** Summary of included studies in the quantitative analysis.

Author, Year	Country	Study Design	Study Population (Criteria)	Inclusion/Exclusion Criteria	Primary and Secondary Outcomes	Type of Vaccine/No of Doses	Time Points of Analysis	Adjusted Vaccine Efficacy/Effectiveness	
								1st Dose	2nd Dose
Baden, 2021 [37]	USA	RCT	Sample size = (30,420) Intervention = (14,550) Control = (14,598) Mean age = (51.4 years) Sex = (47.3% F)	Inclusion: -Age ≥ 18 years. -No known history of SARS-CoV-2. -Absence of SARS-CoV-2–binding antibodies.	Prevention of COVID-19 illness ≥ 14 days after the 2nd dose. Prevention of severe COVID-19 or efficacy of the vaccine at preventing COVID-19 after a single dose or at preventing COVID-19 according to a secondary (CDC, less restrictive case	Moderna/2 doses	14 days after the 1st dose	94.10% for symptomatic infection	
Polack, 2021 [42]	USA	RCT	Sample size = (37,706)	Inclusion: -Age ≥ 16 years, healthy or had stable chronic medical conditions -No medical history of COVID-19 -Did not receive immunosuppressive therapy -Had immunocompromising condition.	Prevention of COVID-19 illness ≥ 7 days after the 2nd dose. Efficacy in participants ± evidence of prior infection	Pfizer–BioNTech/2 doses	≥7 days after the 2nd dose		94.6%
Voysey, 2021a [43]	UK, Brazil, and South Africa	RCT	Sample size = (17,178) Intervention = (8597) Control = (8581)	Inclusion: - Individuals ≥ 18 years	Virologically confirmed symptomatic COVID-19 disease more than 14 days after the 2nd dose. COVID-19 infection at least 22 days after the 1st dose	ChAdOx1 nCoV-19/AstraZeneca/2 doses	3 months after the 1st dose and 14 days after the 2nd dose	76.0%	66.7%
Shinde, 2020 [6]	Saudi Arabia	RCT	Sample size = (4387) Intervention = (2199) Control = (2188)Mean age = (32.0 years) Sex = (43% F)	Inclusion: -Age > 18–84 years, without human immunodeficiency virus infection or a subgroup aged 18–64 years with medically stable HIV. Exclusion: -Pregnancy -Receipt of immunosuppressive therapy autoimmune or immunodeficiency disease -A history of confirmed or suspected COVID-19, and SARS-CoV-2 infection as confirmed on a nucleic acid amplification test (NAAT).	Safety and vaccine efficacy against laboratory-confirmed symptomatic COVID-19 at 7 days or more after the 2nd dose among participants without previous SARS-CoV-2 infection	Novavax/2 doses	≥7 days after the 2nd dose		60.1%
Emary, 2021 [38]	UK	RCT	Sample size = (8534) Intervention = (4244) Control = (4290) Sex = (59% F)	Inclusion: -Age ≥ 18 years who were enrolled in phase 2/3 vaccine efficacy studies in the United Kingdom (UK), and who were randomly assigned (1:1) to receive ChAdOx1 nCoV-19 or a meningococcal conjugate control (MenACWY) vaccine, -people who received 2 doses of the intervention. Exclusion: -Single-dose recipients were excluded. -Cases were excluded if they occurred <15 days after the 2nd dose of vaccine or occurred in participants who were not seronegative on a SARS-CoV-2 N protein assay at baseline.	Symptomatic COVID-19 disease, defined as a positive NAAT result on an upper airway swab in a participant with at least one symptom, including cough, fever of 37.8 °C or higher, shortness of breath, anosmia, or ageusia. The efficacy analysis included symptomatic COVID-19 in seronegative participants with a NAAT positive swab > 14 days after the 2nd dose of vaccine	ChAdOx1 nCoV-19/AstraZeneca/2 doses	≥7 days after the 2nd dose		70.4% for B.1.1.7 and 81.5% for non-B.1.1.7 lineages
Logunov, 2021 [40]	Russia	RCT	Sample size = (19,866 Intervention = (14,964) Control = (4902) [46] Mean age (45·3 years) Sex (38.9% F)	Inclusion: -Age ≥ 18 years -Negative HIV, hepatitis B and C, and syphilis test results. Exclusion: -known history of SARS-CoV-2 -Positive drug and alcohol tests at screening visit -History of vaccine-induced reactions. -Pregnancy or breastfeeding. -The active form of a disease caused by HIV, syphilis, or hepatitis B or C.	Confirmed COVID-19 by PCR from day 21 after receiving the 1st dose	Gam-COVID-Vac/2 doses	≥7 days after the 1st dose.	91.6%	
Kaabi, 2021 [36]	UAE and Bahrain	RCT	Sample size = (40,382) (13,459 Received SARS-CoV-2WIV04 and 13,465 received HB02) Control = (13, 458) Mean age = (36.1 years) Sex = (15.6% F)	Inclusion: -Age ≥ 18 years -Non-pregnant -With self-ability to understand the study procedures and sign the informed consent form. Exclusion: -Confirmed acute cases of SARS-CoV-2 infection -a medical history of SARS, MERS virus infection -with severe chronic illness and other circumstances judged by investigators	Symptomatic laboratory-confirmed COVID-19 case that occurred ≥ 14 days after 2nd dose	SARS-CoV-2 WIV04/2 doses and HB02/2 doses	≥7 days after the 2nd dose		72.8%for SARS-CoV-2 WIV04 and 78.1% for HB02
Frenck, 2021 [39]	Multinational	RCT	Sample size = (2260) Intervention = (1131) Control = (1129) Sex = (49.9% F)	Inclusion: -Age 12–15 years -Healthy or had stable pre-existing disease (hepatitis B, hepatitis C, or HIV. Exclusion: -History of SARS-CoV-2 infection -Had immunocompromising or immunodeficiency disorder, or treatment with immunosuppressive therapy.	Safety objectives included the assessment of local or systemic reactogenicity events, immunogenicity assessments (SARS-CoV-2 serum neutralization assay, and receptor-binding domain (RBD). The efficacy of BNT162b2 against confirmed COVID-19 with an onset 7 or more days after dose 2	BNT162b2/2 doses	≥7 days after the 2nd dose		100%
Sadoff, 2021 [30]	Multinational	RCT	Sample size = (39,321) Intervention = (19,630) Control = (19,691)	Inclusion: -Body Mass Index (BMI) <30 kg/m^2^, -Healthy or had stable pre-existing disease (including hepatitis B, hepatitis C, or HIV infection). Exclusion: -History of anaphylaxis or other serious adverse drug reaction (ADR) to vaccines or their excipients. -has an abnormal function of the immune system resulting from clinical conditions -Persons with a previous clinical or virologic COVID-19 diagnosis or SARS-CoV-2 infection	Vaccine efficacy against moderate to severe–critical COVID- 2019) with an onset ≥14 days and ≥28 days after administration	Ad26.COV2. S/1 dose	After the 1st dose	66.9%	
Dagan, 2021 [46]	Israel	Observational study	Sample size = (71,152)	Inclusion: -Age ≥ 16 Y -No known history of SARS-CoV-2 -A member of the health care organization during the previous year. -No Clalit membership (health service organization) -Unmapped place of residence -Being health care workers, and residence in a long-term care facility.	Documented SARS-CoV-2 infection, symptomatic COVID-19, hospital admission for COVID-19, and death from COVID-19	BNT162b2/2 doses	days 14 through 20 (after 1st dose. 7 days after the 2nd dose	57% for symptomatic infection, 74% for hospitalization	94% for symptomatic infection, 87% for hospitalization
Martínez-Baz, 2021 [53]	Spain	Prospective cohort	Sample size = (20,961) (801 received Comirnaty, 524 (Vaxzevria and 56 Moderna vaccine)	Inclusion: -Age ≥ 18 years covered by the Navarre Health Service. -Had been close contacts of laboratory-confirmed -COVID-19 cases from January to April 2021. Exclusion: -Close contacts with a positive test for SARS-CoV-2 before January 2021 -Nursing home residents and those who did not complete the testing protocol	Preventing severe SARS-CoV-2infections, symptomatic confirmed SARS-CoV-2 infections, and COVID-19 hospitalizations	Comirnaty/2 doses, Vaxzervria/2 doses and Moderna/2 doses	After the 1st and 2nd doses	35% for infection, 42% for symptomatic infection, and 72% for hospitalization	66% for infections and 82% for symptomatic infection, and 95% for hospitalization
Swift, 2021 [55]	USA	Retrospective cohort	Sample size = (71,152) Cohort = (47,221)Controls = (23,931)	Inclusion: -Health care workers (HCWs). Exclusion: -Individuals with a positive molecular assay prior to 1 Januaray 2021 or inactive employment status.	Vaccine effectiveness in the subpopulation of health care personnel (HCP) reached through employer vaccination programs	BNT162b2/2 doses and Moderna/2 doses	After the 1st and 2nd doses		96%
Tenforde, 2021 [56]	USA	Case control	Sample size = (417) Cases = (187) Control = (230)	Inclusion: -Age ≥ 65 years -SARS-CoV-2 negative Exclusion: -Participants with unverified vaccination status -Vaccination with Janssen COVID-19 vaccine.	Effectiveness of Pfizer and Moderna vaccines among adults aged >65 years	BNT162b2/2 doses and Moderna/2 doses	After the 1st and 2nd doses	64%	94%
Khan, 2021 [51]	USA	Retrospective cohort	Sample size (14,697) Cohort (7321) Controls (7376)	Inclusion: -Age ≥ 18 years confirmed negative SARS-CoV-2 infection. -who were taking an inflammatory bowel disease (IBD) medication, and who had ≥ 6 months of Veterans Health Administration outpatient visit data prior to the index date. Exclusion: -Janssen COVID-19 vaccine.	Time to SARS-CoV-2 infection, determined by PCR testing. All-cause mortality and severe SARS-CoV-2 infection	BNT162b2/2 doses and Moderna/2 doses	>7 days after the 2nd dose		80.4%
Bernal, 2021 [52]	UK	Case control	Sample size = (156,930) Cases = (138,869) Controls = (18,061)	Inclusion: -Age ≥ 70 years and older who reported symptoms of COVID-19 between 8 December 2020and 19 February 2021 and were successfully linked to vaccination data in the National Immunization Management System. Exclusion: -Those with a previous positive PCR or antibody test result at any time	Confirmed symptomatic SARS-CoV-2 infections. Admissions to hospital for COVID-19, and deaths with COVID-19	BNT162b2/2 doses and ChAdOx1-S)/2 doses	≥42 days after the 1st and 14 after the 2nd dose	60–70% for BNT162b2 and 60–75% for ChAdOx1-S after the 1st dose	89% For BNT162b2
Fabiani, 2021 [47]	Italy	Retrospective cohort	Sample size = (6423) Cases = (5333) Controls = (1090)	Inclusion: -HCWs. -HCWs infected with COVID-19 before the vaccination campaign, -HCWs working outside hospitals and district outpatient centers, support staff, and administrative staff.	Effectiveness of the vaccine	BNT162b2/2 doses	14–21 days After 1st dose and ≥7 days after 2nd dose	84%	95%
Gras-Valentí, 2021 [48]	Spain	Case control	Sample size = (268) Cases = (70) Controls = (198)	Inclusion: -HCP with suspected COVID-19 and HCP close contacts of COVID-19 cases were included and PCR tested for COVID-19; those with positive PCR were considered cases, and those with negative PCR were considered controls.	Effectiveness of a dose of (BNT162b2) after 12 days of administration in health personnel of a department of Health	BNT162b2/1 dose	12 days After the 1st dose	52.6%, the adjusted vaccine efficacy was 74.6%	
Hall, 2021 [50]	UK	Prospective cohort	Sample size = (1,106,905) Cohort = (396,318) Control = (710,587) Median age = (46·1 years) Sex (84% F)	Inclusion: -HCWs, support staff, and administrative staff (aged ≥18 years) working at hospital sites participating in SIREN who could provide written informed consent and anticipated remaining engaged in follow-up for 12 months. Exclusion: -Had no PCR tests after 7 December 2020, or had insufficient PCR and antibody to complete cohort assignment	Vaccinated participants for the vaccine coverage analysis and SARS-CoV-2 infection confirmed by a PCR test for the vaccine effectiveness analysis	BNT162b2/2 doses	After the 1st and 2nd doses	70%	85%
Haas 2021 [49]	Israel	Observational study	Sample size = (6,538,911) Vaccinated = (4,714,932) Controls = (1,823,979)	Inclusion: -Residents of Israel who aged ≥16 years. Exclusion: -Had received only one dose or had received two doses of BNT162b2 and fewer than 7 days had passed since the 2nd dose.	COVID-19 infection Hospitalization, severe infection, symptomatic infection, and death	BNT162b/2 doses	>7 days after the 2nd dose		91.5% for asymptomatic infection, 97.0% for symptomatic COVID-19 and 96.7% against COVID-19-related death
Pilishvili, 2021 [14]	USA	Cases-control	Sample size = (1843) Cases = (623) Controls = (1220)	Inclusion: -HCWs with positive SARS-CoV-2 test symptoms were enrolled as case-patients, and HCWs with negative SARS-CoV-2 PCR test results, regardless of symptoms, were eligible for enrolment as controls. Exclusion: -Positive SARS-CoV-2 PCR or antigen-based test result >60 days earlier		BNT162b2/2 doses and Moderna/2 doses	14 days after the 1st dose and ≥7 days after the 2nd dose	82%	94%
Vasileiou, 2021 [57]	UK	Prospective cohort	Sample size = (4,409,588) Cohort = (1,331,993) Controls = (3,077,595)	Inclusion: -Received a single dose of the vaccine between 8 December 2020 and 22 February 2021, with maximum follow-up time censored on 22 February 2021. Exclusion: -Previously tested positive with real-time reverse transcription-PCR (RT- PCR) for SARS-CoV-2 infection.	Any hospital admission with COVID-19 as the main cause, or hospital admission within 28 days of a positive RT-PCR test for SARS-CoV-2 infection from 8 December 2020 to 22 February 2021	BNT162b2/2 doses and ChAdOx1-S)/2 doses	28–34 days post the 1st dose vaccination.	91% for BNT162b2 and 88% for the ChAdOx1 vaccine	
Britton, 2021 [45]	USA	Retrospective cohort	Sample size (463) (304 had received 2 doses of vaccine, 72 had received 1 dose and 87 had not received any doses).	Inclusion: -Residents of skilled nursing facilities were included if they were admitted at either facility during one or more rounds of facility-wide SARS-CoV-2 testing during the week before or any time after their facility’s first vaccination clinic.		BNT162b2/2 doses	After the 1st dose	63%	
Madhi, 2021 [41]	South Africa	RCT	Sample size: 2026 Intervention (1013) Control (1013) Median age: 30 years Sex: 56.5% M	HIV-negative adults aged 18 to >65 years. HIV-positive adults at screening, previous or current laboratory- confirmed COVID-19, a history of anaphylaxis in relation to vaccination, and morbid obesity (BMI ≥ 40).	Safety and efficacy of the vaccine against laboratory-confirmed symptomatic COVID-19 more than 14 days after the 2nd dose	ChAdOx1/2 doses	14 days after the 2nd dose		21.9%
Menni, Klaser, 2021 [54]	UK	Prospective observational study	Sample Size (67,293 users received BNT162b2 and 36,329 received ChAdOx1 nCoV-19 Compared to 464,356 unvaccinated users)	Not reported.	The proportion of app users reporting adverse effects within 8 days after vaccination. Infection rates in individuals after receiving the 1st dose of either the vaccines	BNT162b2/2 doses and ChAdOx1-S)/2 doses	8 days after the 1st dose	64% for BNT162b2 and 52% for ChAdOx1	
Voysey, 2021b [44]	UK, Brazil, and South Africa	RCT	Sample size COV002 (UK; LD/SD; n = 2741) COV002 (UK; SD/SD; n = 4807) COV003 (Brazil; all SD/SD; n = 4088)	Inclusion: -The first symptom or first NAAT-positive result was on or before the data cutoff date (4 November 2020). Exclusion: -Seropositive participants at baseline or those who had no baseline result were excluded. -NAAT-positive swabs within 14 days after the 2nd dose.	Virologically confirmed, symptomatic/asymptomatic COVID-19 infection, in COV002 in UK tested weekly by self-administered nose and throat swab from 1 week after 1st dose of vaccination using kits provided Adverse events	COV002: LD (2.2 × 10¹⁰ viral particles), SD (5 × 10¹⁰ viral particles) COV003/2 doses of the vaccine at a dose of 3.5–6.5 × 10¹⁰ viral particles with administration up to 12 weeks apart (target 4 weeks)	>14 days after their 2nd dose		70.4%

F = females; NAAT, a nucleic acid amplification test; HIV = human immunodeficiency virus; SARS = severe acute respiratory syndrome; MERS = Middle East respiratory syndrome; RBD = receptor-binding domain; ADR = serious adverse drug reaction; BMI = body mass index; HCP = health care personal; IBD = inflammatory bowel disease; VHA = Veterans Health Administration; USA = United States of America; UK = United Kingdom; UAE = United Arab Emirates.

## Data Availability

The data presented in this study are available on request from the corresponding author.

## Supplementary Materials

The following supporting information can be downloaded at: https://www.mdpi.com/article/10.3390/vaccines10030350/s1, Table S1: PRISMA 2020 Checklist. Table S2: Summary of studies not met inclusion criteria of qualitative and quantitative analysis. Table S3: Quality Assessment Tool for Observational Cohort and Cross-Sectional Studies.
