# Supplementary material for: Efficacy and Effectiveness of SARS-CoV-2 Vaccines: A Systematic Review and Meta-Analysis
Source: Vaccines (Basel). 2022 Feb 23;10(3):350. doi: 10.3390/vaccines10030350 (PMC8948677; doi:10.3390/vaccines10030350)
Supplement: Supplementary file 1 [file vaccines-10-00350-s001.zip › vaccines-1569748-supplementary.pdf]

Supplementary

Table S1: PRISMA 2020 Checklist [1]

Section and Topic	Item #	Checklist item	Location where item is reported
**TITLE**			
Title	1	Identify the report as a systematic review.	P1
**ABSTRACT**			
Abstract	2	See the PRISMA 2020 for Abstracts checklist.	P2
**INTRODUCTION**			
Rationale	3	Describe the rationale for the review in the context of existing knowledge.	P3
Objectives	4	Provide an explicit statement of the objective(s) or question(s) the review addresses.	P4
**METHODS**			
Eligibility criteria	5	Specify the inclusion and exclusion criteria for the review and how studies were grouped for the syntheses.	P4
Information sources	6	Specify all databases, registers, websites, organisations, reference lists and other sources searched or consulted to identify studies. Specify the date when each source was last searched or consulted.	P5
Search strategy	7	Present the full search strategies for all databases, registers and websites, including any filters and limits used.	P5-6
Selection process	8	Specify the methods used to decide whether a study met the inclusion criteria of the review, including how many reviewers screened each record and each report retrieved, whether they worked independently, and if applicable, details of automation tools used in the process.	P6
Data collection process	9	Specify the methods used to collect data from reports, including how many reviewers collected data from each report, whether they worked independently, any processes for obtaining or confirming data from study investigators, and if applicable, details of automation tools used in the process.	P7
Data items	10a	List and define all outcomes for which data were sought. Specify whether all results that were compatible with each outcome domain in each study were sought (e.g. for all measures, time points, analyses), and if not, the methods used to decide which results to collect.	P6
	10b	List and define all other variables for which data were sought (e.g. participant and intervention characteristics, funding sources). Describe any assumptions made about any missing or unclear information.	P6
Study risk of bias assessment	11	Specify the methods used to assess risk of bias in the included studies, including details of the tool(s) used, how many reviewers assessed each study and whether they worked independently, and if applicable, details of automation tools used in the process.	P6
Effect measures	12	Specify for each outcome the effect measure(s) (e.g. risk ratio, mean difference) used in the synthesis or presentation of results.	P7
Synthesis methods	13a	Describe the processes used to decide which studies were eligible for each synthesis (e.g. tabulating the study intervention characteristics and comparing against the planned groups for each synthesis (item #5)).	P7
	13b	Describe any methods required to prepare the data for presentation or synthesis, such as handling of missing summary statistics, or data conversions.	P6
	13c	Describe any methods used to tabulate or visually display results of individual studies and syntheses.	P6
	13d	Describe any methods used to synthesize results and provide a rationale for the choice(s). If meta-analysis was performed, describe the model(s), method(s) to identify the presence and extent of statistical heterogeneity, and software package(s) used.	P6

Section and Topic	Item #	Checklist item	Location where item is reported
	13e	Describe any methods used to explore possible causes of heterogeneity among study results (e.g. subgroup analysis, meta-regression).	P6
	13f	Describe any sensitivity analyses conducted to assess robustness of the synthesized results.	P6
Reporting bias assessment	14	Describe any methods used to assess risk of bias due to missing results in a synthesis (arising from reporting biases).	P6
Certainty assessment	15	Describe any methods used to assess certainty (or confidence) in the body of evidence for an outcome.	P7
**RESULTS**			
Study selection	16a	Describe the results of the search and selection process, from the number of records identified in the search to the number of studies included in the review, ideally using a flow diagram.	Fig1
	16b	Cite studies that might appear to meet the inclusion criteria, but which were excluded, and explain why they were excluded.	Supplementary 2
Study characteristics	17	Cite each included study and present its characteristics.	P8-18
Risk of bias in studies	18	Present assessments of risk of bias for each included study.	P19 - supplementary 1
Results of individual studies	19	For all outcomes, present, for each study: (a) summary statistics for each group (where appropriate) and (b) an effect estimate and its precision (e.g. confidence/credible interval), ideally using structured tables or plots.	P19-30
Results of syntheses	20a	For each synthesis, briefly summarise the characteristics and risk of bias among contributing studies.	P19-30
	20b	Present results of all statistical syntheses conducted. If meta-analysis was done, present for each the summary estimate and its precision (e.g. confidence/credible interval) and measures of statistical heterogeneity. If comparing groups, describe the direction of the effect.	P19-30
	20c	Present results of all investigations of possible causes of heterogeneity among study results.	P19-30
	20d	Present results of all sensitivity analyses conducted to assess the robustness of the synthesized results.	P19-30
Reporting biases	21	Present assessments of risk of bias due to missing results (arising from reporting biases) for each synthesis assessed.	P19-30
Certainty of evidence	22	Present assessments of certainty (or confidence) in the body of evidence for each outcome assessed.	P19-30
**DISCUSSION**			
Discussion	23a	Provide a general interpretation of the results in the context of other evidence.	P31-32
	23b	Discuss any limitations of the evidence included in the review.	P32
	23c	Discuss any limitations of the review processes used.	P32
	23d	Discuss implications of the results for practice, policy, and future research.	P32
**OTHER INFORMATION**			
Registration and	24a	Provide registration information for the review, including register name and registration number, or state that the review was not registered.	P1

Section and Topic	Item #	Checklist item	Location where item is reported
protocol	24b	Indicate where the review protocol can be accessed, or state that a protocol was not prepared.	P1
	24c	Describe and explain any amendments to information provided at registration or in the protocol.	P1
Support	25	Describe sources of financial or non-financial support for the review, and the role of the funders or sponsors in the review.	P33
Competing interests	26	Declare any competing interests of review authors.	P33
Availability of data, code and other materials	27	Report which of the following are publicly available and where they can be found: template data collection forms; data extracted from included studies; data used for all analyses; analytic code; any other materials used in the review.	P5-P18

Table S2: summary of studies not met inclusion criteria of qualitative and quantitative analysis.

Title	Author	Country	Type of vaccine	Type of study	Reason of rejection	Outcome
A preliminary report of a randomized controlled phase 2 trial of the safety and immunogenicity of mRNA-1273 SARS-CoV-2 vaccine [2]		US	mRNA-1273	RCT	measure safety and immunogenicity	the incidence of symptomatic cases of COVID-19 disease confirmed by RT-PCR two weeks after the second dose of vaccination
Antibody response to inactivated COVID-19 vaccine (CoronaVac) in immune-mediated diseases: a controlled study among hospital workers and elderly[3]		Turkey	Corona-vac	cross sectional	test immunogenicity	Patients with immune mediated disease were significantly less likely to have detectable antibodies than healthy controls both among the hospital workers and elderly. Among patients with IMD, those using immunosuppressive or immune-modulating drugs were significantly less likely to have detectable antibodies compared to those off treatment
BNT162b2 mRNA Covid-19 Vaccine Effectiveness among Health Care Workers [4]		Israel	Pfizer–BioNTech vaccine	cross-sectional	letter to editor	80% reduction of new case post complete vaccination
ChAdOx1 nCoV-19 vaccine for SARS-CoV-2 [5]		India	(ChAdOx1 nCoV-19)	letter to editor	safety and immunogenicity	malaise and myalgia were predictive of allocation to the experimental group
Effectiveness of SARS-CoV-2 vaccination in fully-vaccinated solid organ transplant recipients [6]		US	Pfizer–BioNTech vaccine	A retrospective, observational study	letter to editor no control arm	≤1 dose = 9(1.6%) tested + vet (NAAT) Six of partially vaccinated =98 (6.12%) SOTR developed SARS-CoV-2 infection. positive SARS-CoV-2 in 3 of 459 (0.65%) in fully vaccinated after ≥14 days
Effectiveness of the BNT162b2 Covid-19 Vaccine against the B.1.1.7 and B.1.351 Variants [7]		Qatar	Pfizer–BioNTech vaccine	case-control	letter to editor	PCR-confirmed infection with the B.1.1.7 variant effectiveness<br>After one dose 29.5%<br>≥14 days after second dose 89.5%<br>PCR-confirmed infection with the B.1.351 variant<br>After one dose 16.9%

					≥14 days after second dose 75%<br>Severe, critical, or fatal disease caused by the B.1.1.7 variant effectiveness<br>After one dose 54.1%<br>≥14 days after second dose 100.0<br>Severe, critical, or fatal disease caused by the B.1.351 variant<br>After one dose 0.0<br>≥14 days after second dose 100
Post-vaccination SARS-CoV-2 infections and incidence of the B.1.427/B.1.429 variant among healthcare personnel at a northern California academic medical center [8]	US	Pfizer–BioNTech vaccine	observational	no control	>=23,090 HCPS received ≧1 dose of an mRNA-based SARS-CoV-2 vaccine,<br>660 HCP cases of which 189 were post vaccinated. Among the PVSCs, 114 (60.3%), 49 (25.9%) and 26 (13.8%) were early post-vaccination, partially vaccinated, and fully vaccinated, respectively. Of 261 from vaccinated and unvaccinated HCP,<br>103 (39.5%), including 42 PVSCs (36.5%), had L452R mutation presumed to be B.1.427/B.1.429.
Prior COVID-19 Infection and Antibody Response to Single Versus Double Dose mRNA SARS-CoV-2 Vaccination [9]	US	Pfizer–BioNTech vaccine	observational	immunogenicity	a single dose of mRNA vaccine could provoke in COVID-19 recovered individuals a level of immunity that is comparable to that seen in infection naïve persons following a double dose regimen
Reactogenicity, safety and antibody response, after one and two doses of mRNA-1273 in seronegative and seropositive healthcare workers[10]	Belgium	BNT162b2	letter to editor	letter to editor	effect of vaccination on antibody titre and side effect. Two samples were taken from all participants 2 weeks after the first injection (T1) and 2 weeks after the second injection (T2) Except for 2 individuals, all participants who were seropositive at T0, saw their antibody levels boosted by the first dose but no additional boosting effect was observed after the second injection. In these two individuals (1.6%), the second injection made it possible to raise their antibody levels from 59.7 and 105 AU/mL to above the maximum detection limit (> 400 AU/mL) at T2. In seronegative participants, the anti-S antibody titres obtained after a single dose were comparable to those obtained in unvaccinated seropositive participants while the second injection was necessary to achieve higher antibody levels approaching those obtained for seropositive individuals. Common side effects such as articular pain, muscular pain, headache, fatigue, fever, adenopathy and oedema from the first dose appear to be more frequent and severe in previously infected individuals. the second injection generates a greater overall systemic reaction than that observed after the first one, regardless of the initial serological status of the participants

Reduced mortality rate after Coronavac vaccine among healthcare workers [11]	Turkey	Corona-vac	observational study retrospective pre and post vaccination	letter to editor	the ratio of the death of selected healthcare workers to all residents (SEL/ALL) decreased after vaccination.
Reduction in COVID-19 Patients Requiring Mechanical Ventilation Following Implementation of a National COVID-19 Vaccination Program — Israel, December 2020–February 2021 [12]	Israel	Pfizer BioNTech	Ecological study	letter to editor	2-dose vaccination coverage was 84% among persons aged ≥70 years and 10% among those aged <50 years. The ratio of COVID-19 patients aged ≥70 years requiring mechanical ventilation to those aged <50 years declined 67%
Safety and efficacy of the BNT162b2 mRNA Covid-19 vaccine during Ixekizumab treatment for hidradenitis suppurativa [13]	US	Pfizer BioNTech	Case report	letter to editor	vaccine administration in a patient with HS treated with anti–IL-17 drugs is safe and effective. The patient developed high-titled antibody response,
Safety of New mRNA Vaccines Against COVID-19 in Severely Allergic Patients [14]	Spain	(Pfizer BioNTech) [1], and COVID-19 Vaccine Moderna® (Moderna)	Cross sectional study	Side effect of vaccine with no control	subjects received the first dose of a mRNA COVID-19 vaccine. There was only a mild immediate reaction in a 43-year-old woman with a personal history of severe asthma, 10 minutes after the administration of the BioNTech Pfizer mRNA vaccine. S
Safety and immunogenicity of COVID-19 vaccination in patients with non-alcoholic fatty liver disease (CHESS2101): A multicentre study [15]	China	inactivated vaccine against SARS-CoV-2	Multicentred study	adverse reaction	The number of adverse reactions within 7 days after each injection and adverse reactions within 28 days total 95 (24.9%) and 112 (29.4%), respectively. The most common adverse reactions were injection site pain in 70 (18.4%), followed by muscle pain in 21 (5.5%), and headache in 20 (5.2%). All adverse reactions were mild and self-limiting, and no grade 3 adverse reactions were recorded. Notably, neutralizing antibodies against SARS-CoV-2 were detected in 364 (95.5%) patients with NAFLD. The median neutralizing antibody titre was 32 (IQR 8-64), and the neutralizing antibody titres were maintained.
SARS-CoV-2 Vaccine-Induced Immune Thrombotic Thrombocytopenia (Apr, 10.1056/NEJMe2106315, 2021) [16]	Norway	ChAdOx1 nCoV-19 (AstraZeneca)	editorial article	editorial article	Levels of d-dimer were elevated at the time of admission in all patients. The international normalized ratio (INR) and activated partial thromboplastin time were within the normal range. The fibrinogen level was lower than normal in Patient 2 and was slightly lower than normal in Patients 4 and 5 The C-reactive protein level was moderately elevated in Patients 1, 3, and 5. Screening for thrombophilia with proteins C and S and antithrombin was negative. Antiphospholipid antibodies were detected only in patient 3, who had a slightly elevated anticardiolipin IgG antibody level of 43 IgG phospholipid (GPL) units. Levels of complement proteins (C1q, C4, and C3) and activation products (sC5b-9) were within the normal range in all patients. No patients had signs of haemolysis, and ADAMTS13 activity

					was normal in the one patient in whom it was assessed.
Serological response to COVID-19 vaccination in patients with cancer older than 80 years [17]	Italy	BNT162b2 vaccine.	control trial	serological response	patients with cancer aged ≥80 years can have a serological response to the BNT162b2 COVID-19 vaccine one month after vaccination
Serum neutralising activity against SARS-CoV-2 variants elicited by CoronaVac [18]	China	Sinovac Biotech	observational study	serological response	All pre-vaccine sera showed undetectable levels of neutralisation against the seven SARS-CoV-2 variants tested. After two-dose inoculation, 76 (82%; geometric mean titre [GMT] 51·3) serum samples were capable of neutralising wildtype pseudo virus.
Serum Neutralizing Activity Elicited by mRNA-1273 Vaccine [19]	UK	mRNA-1273 vaccine	observational study	letter to editor no control arm	assessed neutralisation activity
Serum sample neutralisation of BBIBP-CorV and ZF2001 vaccines to SARS-CoV-2 501Y.V2 [20]	China	BBIBP-CorV and BBIBP-CorV and	observational study	letter to editor no control arm	Study findings suggest that the 501Y.V2 variant does not escape the immunity induced by vaccines targeting the whole virus (BBIBP-CorV) or S protein dimeric RBD (ZF2001). The potential 1·5 to 1·6 times reduction in neutralising GMTs should be taken into account for their effect on the clinical efficacy of these vaccines. For both vaccines, immune serum samples neutralise both variant 501Y.V2 and D614G, the variant currently circulating globally, non-significantly
Severe acute respiratory syndrome coronavirus 2 (SARS-CoV-2) infection in vaccinated and unvaccinated healthcare personnel in a Veterans' Affairs healthcare system [21]	US	BNT162b2	observational study	letter to editor	vaccinated personnel were 19 times less likely than unvaccinated personnel to be diagnosed with COVID-19. The percentage of infections with the B.1.1.7 variant among vaccinated personnel was equivalent to the overall percentage of B.1.1.7 infections in our facility. No other currently classified variants of concern or interest were detected among the breakthrough infections. Most SARS-CoV-2 infections in vaccinated personnel occurred after higher-risk exposures to family members
Short-term safety of the BNT162b2 mRNA COVID-19 vaccine in patients with cancer treated with immune checkpoint inhibitors [22]	Israel	Pfizer BNT162b2 mRNA vaccine	observational study	article	The side-effect profile was similar in the healthy controls and the patients with cancer, apart from muscle pain, which was significantly more common among patients with cancer. However, no immune-related myositis was diagnosed after the vaccine in either patients or controls. This observation further supports the safety of the BNT162b2 mRNA vaccine in patients with cancer being treated with immune checkpoint inhibitors.
Side effects of BNT162b2 mRNA COVID-19 vaccine: A randomized, cross-sectional study with detailed self-reported symptoms from healthcare workers [23]	US	BNT162b2 mRNA vaccine	A randomized, cross-sectional study	safety and side effect	Commonly reported symptoms (occurrence in descending order) were soreness, fatigue, myalgia, headache, chills, fever, joint pain, nausea, muscle spasm, sweating, dizziness, flushing, feelings of relief, brain fogging, anorexia, localized swelling, decreased sleep quality, itching, tingling, diarrhoea, nasal stuffiness, and palpitations. Despite this, remarkable acceptance for the second dose of the BNT162b2 vaccine was found among HCWs.

**Single-dose BNT162b2 mRNA COVID-19 vaccine significantly boosts neutralizing antibody response in health care workers recovering from asymptomatic or mild natural SARS-CoV-2 infection** [24]	**Italy**	BNT162b2 mRNA	**case control**	**immunogenicity**	**A single vaccination in people with mild or asymptomatic previous infection further boosts SARS-CoV-2 humoral immunity to levels higher than those obtained by complete two-vaccination in uninfected subjects.**
**Sputnik V COVID-19 vaccine candidate appears safe and effective[25]**		**AstraZeneca ChAdOx1 nCoV-19**	**review article**	**review article no control group**	**62 (1·3%) of 4902 individuals in the placebo group and 16 (0·1%) of 14 964 participants in the vaccine group had confirmed SARS-CoV-2 infection from day 21 after first vaccine dose (the primary outcome). A time-resolved plot of the incidence rate in the two groups showed that the immunity required to prevent disease arose within 18 days of the first dose. the severity of disease decreases as immunity develops. serious adverse events unrelated to the vaccine were reported in 45 participants from the vaccine group and 23 participants from the placebo group. Vaccine efficacy, based on the numbers of confirmed COVID-19 cases from 21 days after the first dose of vaccine, is reported as 91·6% (95% CI 85·6–95·2), and the suggested lessening of disease severity after one dose is particularly encouraging for current dose-sparing strategies.**
**The Gender Impact Assessment among Healthcare Workers in the SARS-CoV-2 Vaccination—An Analysis of Serological Response and Side Effects** [26]	**Italy**	**BNT162b2 mRNA**	**cross sectional**	**immunogenicity**	**Nine percent of cases (5.4% females and 3.4% males) were positive at T_0, indicating a natural immune response to SARS-CoV-2 in these subjects. 98.2% had reactivity in response to the first vaccine dose, while only 55 subjects (1.8% of T_0 seronegative individuals) showed an antibody titre below the cut-off threshold value, with a statistically significant difference between males (2.6%) and females (1.4%). Evaluation of T2 a statistically significant difference was observed among <40 yr males vs. the >60 yr ($p = 0.01$) and the 40–60 yrs. ($p = 0.036$) age groups. Furthermore, the younger females (<48 yr) showed a significantly higher antibody titre than the 48–52 yrs. subjects ($p = 0.014$) and the >52 yr ones ($p < 0.001$). Interestingly, the overall serological values were significantly higher in females than in males. Males and females showed similar incidences in terms of allergic reactions after the first dose while they were more frequent in the female subjects after the second dose Local reactions had a similar incidence in males and females both after the first and the second dose.**
**The initial impact of a national BNT162b2 mRNA COVID-19 vaccine rollout** [27] **Qatar**		**BNT162b2**	**cross sectional**	**no control**	**A total of 199,219 individuals with 6,521,124 person-days of follow-up were included. SARS-CoV-2 infection was confirmed in 1877 (0.9%), 489 (26.1%) were asymptomatic 123 (6.6%) required oxygen support. The median time from first vaccination to SARS-CoV-2 confirmation was 11.9 days (IQR 7.7–18.2).**

					Compared with the first 7-day post-vaccination period, SARS-CoV-2 infections were lower by 65.8–84.7% during 15–21, 22–28, and >28 days (P < 0.001 for each). For severe COVID-19, the incidence rates were 75.7–93.3% lower during the corresponding time periods (P < 0.001 for each).
Thrombocytopenia following Pfizer and Moderna SARS-CoV-2 vaccination [28]	US	Pfizer and Moderna	case report	report on adverse effect	side effect of thrombocytopenia
Thromboembolic Events in the South African Ad26.COV2. S Vaccine Study [29]	South Africa	Ad26.COV2. S vaccine	letter to editor	letter to editor about side effect	side effect of thromboembolism
Thromboembolism and the Oxford–AstraZeneca COVID-19 vaccine: side-effect or coincidence? [30] Denmark		Oxford–AstraZeneca COVID-19 vaccine	cross sectional	side effect	side effect of thromboembolism
Thrombotic events after AstraZeneca vaccine: What if it was related to dysfunctional immune response? [31] France		AstraZeneca ChAdOx1 nCoV-19	letter to editor	letter to editor about side effect	one hypothesis linking the vaccine to the occurrence of serious thrombosis could be the passage into bloodstream of adenovirus which, in the presence of factors not fully identified, would generate a discordant immune response with platelet activation, potentially associated with a NET effect or a decrease in the level of ACE2 on the surface of endothelial cells, leading to an excessive thrombotic risk.
Weak humoral immune reactivity among residents of long-term care facilities following one dose of the BNT162b2 mRNA COVID-19 vaccine [32]	Canada	BNT162b2 mRNA vaccine		Immunogenicity	After one vaccine dose, binding antibodies against S/RBD were ~4-fold lower in residents compared to HCW. Inhibition of ACE2 binding was 3-fold lower in residents compared to HCW and pseudo virus neutralizing activity was 2-fold lower While six (33%) seronegative HCW neutralized live SARS-CoV-2, only one (8%) resident did. In contrast, convalescent HCW displayed 7- to 20-fold higher levels of binding antibodies and substantial ability to neutralize live virus after one dose.
Single-dose BNT162b2 vaccine protects against asymptomatic SARS-CoV-2 infection [33]	UK	Pfizer-BioNTech BNT162b2 mRNA	cross sectional screening	short report	Twenty-six of 3252 tests from unvaccinated HCWs were positive (Ct < 36), compared to 13/3535 tests from HCWs <12 days post-vaccination and 4/1989aQ tests from HCWs ≥12 days post-vaccination. This suggests a fourfold decrease in the risk of asymptomatic SARS-CoV-2 infection amongst HCWs ≥12 days post-vaccination, compared to unvaccinated HCWs, with an intermediate effect amongst HCWs <12 days post-vaccination.
Impact of first dose of BNT162b2 vaccine on COVID-19 infection among healthcare workers in an Irish hospital		BNT162b2 vaccine	letter to editor	letter to editor	1708 staff (38%) had a COVID-19 test, 1314 (77%) of which were known to have received their first vaccine dose. Positivity rates between the vaccinated and unvaccinated

[34] **Ireland**					**groups differed significantly with 5.8% of the vaccinated cohort testing COVID-19-positive versus 25.6% of those tested in the unvaccinated cohort**
**Effectiveness of BNT162b2 vaccine against the B.1.1.7 variant of SARS-CoV-2 among healthcare workers in Brescia, Italy** [35]	**Italy**	**BNT162b2 vaccine**	**letter to editor**	**letter to editor**	**At the end of the observation period, vaccinated HCWs were at lower infection risk as compared to unvaccinated HCWs (by 2.6-folds), and even to a greater extent (6.2 folds) if compared to the general population.**
**Incident SARS-CoV-2 Infection among mRNA-Vaccinated and Unvaccinated Nursing Home Residents** [36]	**US**	**BNT162b2 vaccine – Moderna vaccine**	**letter to editor**	**letter to editor**	**14,669 residents (80.4%) received the Pfizer–BioNTech vaccine, and 3573 (19.6%) received the Moderna vaccine. Of these 18,242 residents, 13,048 also received the second dose of vaccine. A total of 3990 residents were unvaccinated After receipt of the first vaccine dose, there were 822 incident cases (4.5% of vaccinated residents) within 0 to 14 days and 250 cases (1.4%) at 15 to 28 days. Among the 13,048 residents who received both doses of vaccine, there were 130 incident cases (1.0% of vaccinated residents) within 0 to 14 days after receipt of the second dose and 38 cases (0.3%) after 14 days (which included 19 cases occurring 15 to 21 days after receipt of the second dose) (Fig. S1). Among unvaccinated residents, incident cases decreased from 173 cases (4.3% of unvaccinated residents) within 0 to 14 days after the first vaccination clinic to 12 cases (0.3%) at more than 42 days after the clinic**
**Myanmar's health leaders stand against military rule** [37]	**Myanmar**	**BNT162b2 vaccine**	**letter to editor**	**letter to editor**	**170 SARS-CoV-2 infections among HCWs of which 99 (58%) HCWs reported symptoms and were designated as COVID-19 cases. Of the 170 HCWs who became infected, 89 (52%) were unvaccinated, 78 (46%) tested positive after the first dose, and three (2%) tested positive after the second dose. Among the 125 infections that could be traced, 87 (70%) were community acquired SARS-CoV-2 infection rate of 7·4 per 10 000 person days in unvaccinated HCWs, infection rates were 5·5 per 10 000 person-days and 3·0 per 10 000 person-days on days 1–14 and 15-28 after the first dose of the vaccine, respectively. Adjusted rate reductions of SARS-CoV-2 infections were 30% (95% CI 2–50) and 75% (72–84) for days 1–14 and days 15–28 after the first dose,**

Table S3: Quality Assessment Tool for Observational Cohort and Cross-Sectional Studies

Quality Assessment Tool for Observational Cohort and Cross-Sectional Studies																																											
**Study title**		**1. Was the research question or objective in this paper clearly stated?**			**2. Was the study population clearly specified and defined?**			**3. Was the participation rate of eligible persons at least 50%?**			**4. Were all the subjects selected or recruited from the same or similar populations (including the same time period)? Were inclusion and exclusion criteria for being in the study prespecified and applied uniformly to all participants?**			**5. Was a sample size justification, power description, or variance and effect estimates provided?**			**6. For the analyses in this paper, were the exposure(s) of interest measured prior to the outcome(s) being measured?**			**7. Was the timeframe sufficient so that one could reasonably expect to see an association between exposure and outcome if it existed?**			**8. For exposures that can vary in amount or level, did the study examine different levels of the exposure as related to the outcome (e.g., categories of exposure, or exposure measured as continuous variable)?**			**9. Were the exposure measures (independent variables) clearly defined, valid, reliable, and implemented consistently across all study participants?**			**10. Was the exposure(s) assessed more than once over time?**			**11. Were the outcome measures (dependent variables) clearly defined, valid, reliable, and implemented consistently across all study participants?**			**12. Were the outcome assessors blinded to the exposure status of participants?**			**13. Was loss to follow-up after baseline 20% or less?**			**14. Were key potential confounding variables measured and adjusted statistically for their impact on the relationship between exposure(s) and outcome(s)?**		
	**Author**	extractor	revision 1	revision 2	extractor	revision 1	revision 2	extractor	revision 1	revision 2	extractor	revision 1	revision 2	extractor	revision 1	revision 2	extractor	revision 1	revision 2	extractor	revision 1	revision 2	extractor	revision 1	revision 2	extractor	revision 1	revision 2	extractor	revision 1	revision 2	extractor	revision 1	revision 2	extractor	revision 1	revision 2	extractor	revision 1	revision 2	extractor	revision 1	revision 2
BNT162b2 mRNA	Dagan, 2021	1	Low risl		1	Low risk		0	High risk		1	Low risk		0	High risk		1	Low risk		0	Low risk		0	High risk		CD	Low risk		1	Low risk		1	Low risk		0	High risk		1	Low risk		1	Low risk	[38]

Covid-19 Vaccine in a Nationwide Mass Vaccination Setting																																											
Effectiveness of COVID-19 vaccines in preventing SARS-CoV-2 infection and	Martínez-Baz, 2021	1	Low risk		1	Low risk		1	Low risk		1	Low risk		0	0high risk		1	Low risk		0	High risk		NA	High risk		1	Low risk		1	Low risk		0	High risk		0	High risk		1	Low risk		1	Low risk	[39]

hospitalisation, Navarre, Spain, January to April 2021																																											
Effectiveness of mRNA COVID-19 vaccines against SARS-CoV-2 infection in a cohort of health care	Swift, 2021	1	Low risk		1	Low risk		0	Low risk		1	Low risk		0	High risk		0	Low risk		1	Low risk		0	High risk		0	High risk		0	High risk		0	High risk		CD	High risk		CD	Can’t decid		0	High risk	[40]

personnel																																								
Effectiveness of Pfizer-BioNTech and Moderna Vaccines Against COVID-19 Among Hospitalized Adults Aged ≥65 Years — United States,	Tenforde, 2021	1	Low risk		1	Low risk		1	Low risk		1	Low risk		0	High risk		1	Low risk		CD	High risk		0	High risk		0	High risk		0	High risk		1	Low risk		0	High risk		1	Low risk		0	High risk	[41]

January–March 2021																																											
Effectiveness of SARS-CoV-2 Vaccination in a Veterans Affairs Cohort of Patients With Inflammatory Bowel Disease With	Khan, 2021	1	Low risk		1	Low risk		1	Low risk		1	Low risk		0	High risk		1	Low risk		1	Low risk		1	Low risk		1	Low rsk		1	Low risk		1	Low risk		0	High risk		1	Low risk		1	Low risk	[42]

Diverse Exposure to Immunosuppressive Medications																																											
Effectiveness of the Pfizer-BioNTech and Oxford-AstraZeneca vaccines on covid-19 related	Bernal, 2021	**1**	Low risk		1	Low risk		1	Low risk		1	low risk		0	0high risk		1	Low risk		1	Low risk		1	Low risk		1	Low risk		1	Low risk		1	Low risk		0	0high risk		1	Low risk		1	Low risk	[43]

symptoms, hospital admissions, and mortality in older adults																																								
Effectiveness of the Comirnaty BNT162b2, BioNTechPfizer vaccine in preventing SARS-CoV-2	Fabiani, 2021	1	Low risk		1	Low risk		1	Low risk		1	Low risk		0	High risk		1	Low risk		1	Low risk		1	Low risk		1	Low risk		1	Low risk		1	Low risk		0	High risk		1	Low risk		0	High risk	[44]

infection among health care																																											
Effectiveness of the first dose of BNT162b2 vaccine to preventing covid-19 in health care personnel]	Valenti, 2021	1	Low risk		1	Low risk		1	Low risk		1	Low risk		0	High risk		1	Low risk		0	Low risk		1	Low risk		0	High risk		CD	High risk		1	Low risk		0	High risk		1	Low risk		0	high risk	[45]
COVID-19	Hall, 2021	1	Low risk		1	Low risk		CD	Low risk		1	Low risk		0	High risk		1	High risk		1	Loe risk		0	High risk		1	Low risk		1	Low risk		1	Low risk		0	0high risk		1	1low risk		1	Low risk	[46]

vaccine coverage in health-care workers in England and effectiveness of BNT162b2 mRNA vaccine against infection (SIREN): a prospective, multicentre,																																											

cohort study																																														
Impact and effectiveness of mRNA BNT162b2 vaccine against SARSinfections and COVID-19 cases, hospitalisations, and deaths	Haas, 2021	1	Low risk		1	Low risk		1	Low risk		1	Low risk		0	High risk		0	High risk		1	Low risk		1	High risk		0	High risk		0	High risk		1	Low risk		0	High risk		1	Low risk		0	High risk	[47]

following a nationwide vaccination																																											
Interim Estimates of Vaccine Effectiveness of Pfizer-BioNTech and Moderna COVID-19 Vaccines Amon	Pilishvili, 2021	1	Low risk		1	low		1	low		1	low		0	high		0	high		1	low		1	low		1	low		0	high		1	low		0	high		1	low		0	high	[48]

g Health Care Personnel — 33 U.S. Sites,																																										
Interim findings from first-dose mass COVID-19 vaccination roll-out and COVID-19 hospital admissions in	Vasilieou, 2021	1	low		1	low		1	low		1	low		0	high		1	low		1	low		1	low		1	low		1	low		1	low		0	high		1	low		1	low	[49]

Scotland a national prospective cohort study																																											
Effectiveness of the Pfizer-BioNTech COVID-19 Vaccine Among Residents of Two Skilled Nursing Facilities Ex	Britton, 2021	**1**	Low risk		1	Low risk		1	Low risk		1	Low risk		0	High risk		1	Low risk		1	Low risk		0	High risk		1	Low risk		1	Low risk		1	Low risk		0	High risk		1	Low risk		0	High risk	[50]

Vaccine side-effects and SARS-CoV-2 infection after vaccination in users of the COVID Symptom Study app in the UK a prospective observational study	Menni, 2021	1	low		1	low		1	low		1	low		0	high		0	high		1	low		0	high		0	high		0	high		1	high		0	high		1	low		0	high	[51]

Interim Estimates of Vaccine Effectiveness of BNT162b2 and mRNA-1273 COVID-19 Vaccines in Preventing SARS-CoV-2 Infection Among Healt	Thompson, 2021	1	low		1	low		1	low		1	low		0	high		1	low		1	low		1	low		1	low		0	high		1	low		0	high		1	low		0	high	[52]

h Care Per- sonnel																																											

References

1. Page, M.J.; McKenzie, J.E.; Bossuyt, P.M.; Boutron, I.; Hoffmann, T.C.; Mulrow, C.D.; Shamseer, L.; Tetzlaff, J.M.; Akl, E.A.; Brennan, S.E. The PRISMA 2020 statement: an updated guideline for reporting systematic reviews. *Int.J.Surg.* **2021**, *88*, 105906.
2. Chu, L.; McPhee, R.; Huang, W.; Bennett, H.; Pajon, R.; Nestorova, B.; Leav, B. A preliminary report of a randomized controlled phase 2 trial of the safety and immunogenicity of mRNA-1273 SARS-CoV-2 vaccine. *Vacciness* **2021**, *39*, 2791–2799, doi:10.1016/j.vaccine.2021.02.007.
3. Seyahi, E.; Bakhdiyarli, G.; Oztas, M.; Kuskucu, M.A.; Tok, Y.; Sut, N.; Ozcifci, G.; Ozcaglayan, A.; Balkan, II; Saltoglu, N.; et al. Antibody response to inactivated COVID-19 vaccine (CoronaVac) in immune-mediated diseases: a controlled study among hospital workers and elderly. *Rheumatol Int*. **2021**, *41*, 1429–1440, doi:10.1007/s00296-021-04910-7.
4. Benenson, S.; Oster, Y.; Cohen, M.J.; Nir-Paz, R. BNT162b2 mRNA Covid-19 Vaccine Effectiveness among Health Care Workers. *N. Engl. J. Med.* **2021**, *384*, 1775–1777, doi:10.1056/NEJMc2101951.
5. Chauhan, A.; Agarwal, A.; Jaiswal, N.; Singh, M. ChAdOx1 nCoV-19 vaccine for SARS-CoV-2. *The Lancet* **2020**, *396*, 1485–1486.
6. Malinis, M.; Cohen, E.; Azar, M.M. Effectiveness of SARS-CoV-2 vaccination in fully vaccinated solid organ transplant recipients. *Am. J. Transplant* **2021**, *21*, 2916–2918.
7. Abu-Raddad, L.J.; Chemaitelly, H.; Butt, A.A. Effectiveness of the BNT162b2 Covid-19 Vaccine against the B. 1.1. 7 and B. 1.351 Variants. *N. Engl. J. Med.* **2021**, *385*, 187–189.
8. Jacobson, K.B.; Pinsky, B.A.; Rath, M.E.M.; Wang, H.; Miller, J.A.; Skhiri, M.; Shepard, J.; Mathew, R.; Lee, G.; Bohman, B. Post-vaccination SARS-CoV-2 infections and incidence of the B. 1.427/B. 1.429 variant among healthcare personnel at a northern California academic medical center. *MedRxiv.* **2021**.
9. Ebinger, J.E.; Fert-Bober, J.; Printsev, I.; Wu, M.; Sun, N.; Figueiredo, J.C.; Van Eyk, J.; Braun, J.; Cheng, S.; Sobhani, K. Prior COVID-19 infection and antibody response to single versus double dose mRNA SARS-CoV-2 vaccination. *medRxiv* **2021**.
10. Tré-Hardy, M.; Cupaiolo, R.; Papleux, E.; Wilmet, A.; Horeanga, A.; Antoine-Moussiaux, T.; Della Vecchia, A.; Beukinga, I.; Vekemans, M.; Blairon, L. Reactogenicity, safety and antibody response, after one and two doses of mRNA-1273 in seronegative and seropositive healthcare workers. *J. Infect.* **2021**, *83*, 237–279.
11. Akpolat, T.; Uzun, O. Reduced mortality rate after coronavac vaccine among healthcare workers. *J. Infect.* **2021**, *83*, e20–e21.
12. Rinott, E.; Youngster, I.; Lewis, Y.E. Reduction in COVID-19 patients requiring mechanical ventilation following implementation of a national COVID-19 vaccination program—Israel, December 2020–February 2021. *MMWR.* **2021**, *70*, 326.

13. Iannone, M.; Janowska, A.; Tonini, G.; Davini, G.; Dini, V. Safety and efficacy of the BNT162b2 mRNA COVID-19 vaccine during Ixekizumab treatment for hidradenitis suppurativa. *Clin. Dermatol* **2021**.
14. Rojas-Pérez-Ezquerra, P.; Quirós, J.C.; Molina, P.T.; de Ocáriz, M.B.O.; Ortuño, J.Z. Safety of new mRNA vaccines against COVID-19 in severely allergic patients. *JIACI.* **2021**, *31*, 180–181.
15. Wang, J.; Hou, Z.; Liu, J.; Gu, Y.; Wu, Y.; Chen, Z.; Ji, J.; Diao, S.; Qiu, Y.; Zou, S. Safety and immunogenicity of COVID-19 vaccination in patients with non-alcoholic fatty liver disease (CHESS2101): a multicenter study. *J. Hepatol.* **2021**, *75*, 439–441.
16. Cines, D.B.; Bussel, J.B. SARS-CoV-2 vaccine–induced immune thrombotic thrombocytopenia. *N. Engl. J. Med.* **2021**, *384*, 2254–2256.
17. Iacono, D.; Cerbone, L.; Palombi, L.; Cavalieri, E.; Sperduti, I.; Cocchiara, R.A.; Mariani, B.; Parisi, G.; Garufi, C. Serological response to COVID-19 vaccination in patients with cancer older than 80 years. *J. Geriatr. Oncol.* **2021**, *12*, 1253–1255.
18. Chen, Y.; Shen, H.; Huang, R.; Tong, X.; Wu, C. Serum neutralising activity against SARS-CoV-2 variants elicited by CoronaVac. *Lancet Infect. Dis.* **2021**, *21*, 1071–1072.
19. Wu, K.; Werner, A.P.; Koch, M.; Choi, A.; Narayanan, E.; Stewart-Jones, G.B.; Colpitts, T.; Bennett, H.; Boyoglu-Barnum, S.; Shi, W. Serum neutralizing activity elicited by mRNA-1273 vaccine. *N. Engl. J. Med.* **2021**, *384*, 1468–1470.
20. Huang, B.; Dai, L.; Wang, H.; Hu, Z.; Yang, X.; Tan, W.; Gao, G.F. Serum sample neutralisation of BBIBP-CorV and ZF2001 vaccines to SARS-CoV-2 501Y. V2. *The Lancet Microbe.* **2021**, *2*, e285.
21. Redmond, S.N.; Jones, L.D.; Sadri, N.; Schmotzer, C.; Navas, M.E.; Zabarsky, T.F.; Bhullar, D.; Donskey, C.J. Severe acute respiratory syndrome coronavirus 2 (SARS-CoV-2) infection in vaccinated and unvaccinated healthcare personnel in a Veterans Affairs healthcare system. *Infect Control Hosp. Epidemiol.* **2021**, 1-6.
22. Waissengrin, B.; Agbarya, A.; Safadi, E.; Padova, H.; Wolf, I. Short-term safety of the BNT162b2 mRNA COVID-19 vaccine in patients with cancer treated with immune checkpoint inhibitors. *Lancet Oncol.* **2021**, *22*, 581–583.
23. Kadali, R.A.K.; Janagama, R.; Peruru, S.; Malayala, S.V. Side effects of BNT162b2 mRNA COVID-19 vaccine: A randomized, cross-sectional study with detailed self-reported symptoms from healthcare workers. *IJID.* **2021**, *106*, 376–381, doi:10.1016/j.ijid.2021.04.047.
24. Vicenti, I.; Gatti, F.; Scaggiante, R.; Boccuto, A.; Zago, D.; Basso, M.; Dragoni, F.; Zazzi, M.; Parisi, S.G. Single-dose BNT162b2 mRNA COVID-19 vaccine significantly boosts neutralizing antibody response in health care workers recovering from asymptomatic or mild natural SARS-CoV-2 infection. *IJID.* **2021**, *108*, 176–178, doi:10.1016/j.ijid.2021.05.033.
25. Jones, I.; Roy, P. Sputnik V COVID-19 vaccine candidate appears safe and effective. *The Lancet* **2021**, *397*, 642–643.
26. Di Resta, C.; Ferrari, D.; Viganò, M.; Moro, M.; Sabetta, E.; Minerva, M.; Ambrosio, A.; Locatelli, M.; Tomaiuolo, R. The Gender Impact Assessment among Healthcare Workers in the SARS-CoV-2 Vaccination—An Analysis of Serological Response and Side Effects. *Vaccines* **2021**, *9*, 522.
27. Zaqout, A.; Daghfal, J.; Alaqad, I.; Hussein, S.A.; Aldushain, A.; Almaslamani, M.A.; Abukhattab, M.; Omrani, A.S. The initial impact of a national BNT162b2 mRNA COVID-19 vaccine rollout. *IJID.* **2021**, *108*, 116–118.

28. Lee, E.J.; Cines, D.B.; Gernsheimer, T.; Kessler, C.; Michel, M.; Tarantino, M.D.; Semple, J.W.; Arnold, D.M.; Godeau, B.; Lambert, M.P. Thrombocytopenia following Pfizer and Moderna SARS-CoV-2 vaccination. *Am. J. Hematol.* **2021**, *96*, 534–537.
29. Takuva, S.; Takalani, A.; Garrett, N.; Goga, A.; Peter, J.; Louw, V.; Opie, J.; Jacobson, B.; Sanne, I.; Gail-Bekker, L. Thromboembolic Events in the South African Ad26. COV2. S Vaccine Study. *N. Engl. J. Med.* **2021**, 385-570-571.
30. Østergaard, S.D.; Schmidt, M.; Horváth-Puhó, E.; Thomsen, R.W.; Sørensen, H.T. Thromboembolism and the Oxford–AstraZeneca COVID-19 vaccine: side-effect or coincidence? *The Lancet* **2021**, *397*, 1441–1443.
31. Billy, E.; Clarot, F.; Depagne, C.; Korsia-Meffre, S.; Rochoy, M.; Zores, F. Thrombotic events after AstraZeneca vaccine: What if it was related to dysfunctional immune response? *Therapie.* **2021**, *76*, 367–369.
32. Brockman, M.A.; Mwimanzi, F.; Sang, Y.; Ng, K.; Agafitei, O.; Ennis, S.; Lapointe, H.; Young, L.; Umviligihozo, G.; Burns, L. Weak humoral immune reactivity among residents of long-term care facilities following one dose of the BNT162b2 mRNA COVID-19 vaccine. *medRxiv.* **2021**.
33. Jones, N.K.; Rivett, L.; Seaman, S.; Samworth, R.J.; Warne, B.; Workman, C.; Ferris, M.; Wright, J.; Quinnell, N.; Shaw, A.; et al. Single-dose BNT162b2 vaccine protects against asymptomatic SARS-CoV-2 infection. *Elife* **2021**, *10*, e68808, doi:10.7554/eLife.68808.
34. Walsh, J.; Skally, M.; Traynor, L.; de Barra, E.; Dhuthaigh, A.N.; Hayes, B.; Fitzpatrick, F. Impact of first dose of BNT162b2 vaccine on COVID-19 infection among healthcare workers in an Irish hospital. *Ir. J. Med. Sci.* **2021**, 1-2, doi:10.1007/s11845-021-02658-4.
35. Sansone, E.; Tiraboschi, M.; Sala, E.; Albini, E.; Lombardo, M.; Castelli, F.; De Palma, G. Effectiveness of BNT162b2 vaccine against the B.1.1.7 variant of SARS-CoV-2 among healthcare workers in Brescia, Italy. *J. Infect.* **2021**, *83*, e17-e18, doi:10.1016/j.jinf.2021.04.038.
36. White, E.M.; Yang, X.; Blackman, C.; Feifer, R.A.; Gravenstein, S.; Mor, V. Incident SARS-CoV-2 Infection among mRNA-Vaccinated and Unvaccinated Nursing Home Residents. *The N. Engl. J. Med.* **2021**, *385*, 474–476, doi:10.1056/NEJMc2104849.
37. Soe, Z.W.; Oo, M.M.; Wah, K.S.; Naing, A.T.; Skalicky-Klein, R.; Phillips, G. Myanmar's health leaders stand against military rule. *The Lancet* **2021**, *397*, 875, doi:10.1016/s0140-6736(21)00457-8.
38. Dagan, N.; Barda, N.; Kepten, E.; Miron, O.; Perchik, S.; Katz, M.A.; Hernán, M.A.; Lipsitch, M.; Reis, B.; Balicer, R.D. BNT162b2 mRNA Covid-19 vaccine in a nationwide mass vaccination setting. *N. Engl. J. Med.* **2021**, *384*, 1412–1423.
39. Martínez-Baz, I.; Miqueleiz, A.; Casado, I.; Navascués, A.; Trobajo-Sanmartín, C.; Burgui, C.; Guevara, M.; Ezpeleta, C.; Castilla, J. Effectiveness of COVID-19 vaccines in preventing SARS-CoV-2 infection and hospitalisation, Navarre, Spain, January to April 2021. *Eurosurveillance* **2021**, *26*, 2100438.
40. Swift, M.D.; Breeher, L.E.; Tande, A.J.; Tommaso, C.P.; Hainy, C.M.; Chu, H.; Murad, M.H.; Berbari, E.F.; Virk, A. Effectiveness of mRNA COVID-19 vaccines against SARS-CoV-2 infection in a cohort of healthcare personnel. *Clin. Infect. Dis.* **2021**, *73*, e1376–e1379. doi: 10.1093/cid/ciab361.
41. Tenforde, M.W. Effectiveness of Pfizer-BioNTech and Moderna Vaccines Against COVID-19 Among Hospitalized Adults Aged≥ 65 Years—United States, January–March 2021. *MMWR.* **2021**, *70*.
42. Khan, N.; Mahmud, N. Effectiveness of SARS-CoV-2 vaccination in a Veterans Affairs Cohort of Inflammatory Bowel Disease Patients with Diverse Exposure to Immunosuppressive Medications. *Gastroenterology* **2021**, *161*, 827–836.

43. Bernal, J.L.; Andrews, N.; Gower, C.; Robertson, C.; Stowe, J.; Tessier, E.; Simmons, R.; Cottrell, S.; Roberts, R.; O'Doherty, M. Effectiveness of the Pfizer-BioNTech and Oxford-AstraZeneca vaccines on covid-19 related symptoms, hospital admissions, and mortality in older adults in England: test negative case-control study. *BMJ.* **2021**, *373*.
44. Fabiani, M.; Ramigni, M.; Gobbetto, V.; Mateo-Urdiales, A.; Pezzotti, P.; Piovesan, C. Effectiveness of the Comirnaty (BNT162b2, BioNTech/Pfizer) vaccine in preventing SARS-CoV-2 infection among healthcare workers, Treviso province, Veneto region, Italy, 27 December 2020 to 24 March 2021. *Eurosurveillance* **2021**, *26*, 2100420.
45. Gras-Valentí, P.; Chico-Sánchez, P.; Algado-Sellés, N.; Jiménez-Sepúlveda, N.J.; Gómez-Sotero, I.L.; Fuster-Pérez, M.; Cartagena-Llopis, L.; Sánchez-Valero, M.; Cerezo-Milán, P.; Martínez-Tornero, I. Effectiveness of the first dose of BNT162b2 vaccine to preventing covid-19 in healthcare personnel. *Revista. Espanola de Salud Publica.* **2021**, *95*.
46. Hall, V.J.; Foulkes, S.; Saei, A.; Andrews, N.; Oguti, B.; Charlett, A.; Wellington, E.; Stowe, J.; Gillson, N.; Atti, A. COVID-19 vaccine coverage in health-care workers in England and effectiveness of BNT162b2 mRNA vaccine against infection (SIREN): a prospective, multicentre, cohort study. *The Lancet* **2021**, *397*, 1725–1735.
47. Haas, E.J.; Angulo, F.J.; McLaughlin, J.M.; Anis, E.; Singer, S.R.; Khan, F.; Brooks, N.; Smaja, M.; Mircus, G.; Pan, K. Impact and effectiveness of mRNA BNT162b2 vaccine against SARS-CoV-2 infections and COVID-19 cases, hospitalisations, and deaths following a nationwide vaccination campaign in Israel: An observational study using national surveillance data. *The Lancet* **2021**, *397*, 1819–1829.
48. Pilishvili, T.; Gierke, R.; Fleming-Dutra, K.E.; Farrar, J.L.; Mohr, N.M.; Talan, D.A.; Krishnadasan, A.; Harland, K.K.; Smithline, H.A.; Hou, P.C. Effectiveness of mRNA Covid-19 vaccine among US health care personnel. *N. Engl. J. Med.* **2021**, *385*, e90.
49. Vasileiou, E.; Simpson, C.R.; Shi, T.; Kerr, S.; Agrawal, U.; Akbari, A.; Bedston, S.; Beggs, J.; Bradley, D.; Chuter, A. Interim findings from first-dose mass COVID-19 vaccination roll-out and COVID-19 hospital admissions in Scotland: A national prospective cohort study. *The Lancet* **2021**, *397*, 1646–1657.
50. Britton, A.; Slifka, K.M.J.; Edens, C.; Nanduri, S.A.; Bart, S.M.; Shang, N.; Harizaj, A.; Armstrong, J.; Xu, K.; Ehrlich, H.Y. Effectiveness of the Pfizer-BioNTech COVID-19 vaccine among residents of two skilled nursing facilities experiencing COVID-19 outbreaks—Connecticut, December 2020–February 2021. *MMWR.* **2021**, *70*, 396–401.
51. Menni, C.; Klaser, K.; May, A.; Polidori, L.; Capdevila, J.; Louca, P.; Sudre, C.H.; Nguyen, L.H.; Drew, D.A.; Merino, J. Vaccine side-effects and SARS-CoV-2 infection after vaccination in users of the COVID Symptom Study app in the UK: a prospective observational study. *Lancet Infect. Dis.* **2021**, *21*, 939–949.
52. Thompson, M.G.; Burgess, J.L.; Naleway, A.L.; Tyner, H.L.; Yoon, S.K.; Meece, J.; Olsho, L.E.; Caban-Martinez, A.J.; Fowlkes, A.; Lutrick, K. Interim estimates of vaccine effectiveness of BNT162b2 and mRNA-1273 COVID-19 vaccines in preventing SARS-CoV-2 infection among health care personnel, first responders, and other essential and frontline workers—eight US locations, December 2020–March 2021. *MMWR.* **2021**, *70*, 495–500.
